# Modified carbon nitride nanozyme as bifunctional glucose oxidase-peroxidase for metal-free bioinspired cascade photocatalysis

**DOI:** 10.1038/s41467-019-08731-y

**Published:** 2019-02-26

**Authors:** Peng Zhang, Dengrong Sun, Ara Cho, Seunghyun Weon, Seonggyu Lee, Jinwoo Lee, Jeong Woo Han, Dong-Pyo Kim, Wonyong Choi

**Affiliations:** 1 Division of Environmental Science and Engineering, Pohang University of Science and Technology (POSTECH), Pohang, 37673 Korea; 2 Department of Chemical Engineering, Pohang University of Science and Technology (POSTECH), Pohang, 37673 Korea

## Abstract

Nanomaterials-based biomimetic catalysts with multiple functions are necessary to address challenges in artificial enzymes mimicking physiological processes. Here we report a metal-free nanozyme of modified graphitic carbon nitride and demonstrate its bifunctional enzyme-mimicking roles. With oxidase mimicking, hydrogen peroxide is generated from the coupled photocatalysis of glucose oxidation and dioxygen reduction under visible-light irradiation with a near 100% apparent quantum efficiency. Then, the in situ generated hydrogen peroxide serves for the subsequent peroxidase-mimicking reaction that oxidises a chromogenic substrate on the same catalysts in dark to complete the bifunctional oxidase-peroxidase for biomimetic detection of glucose. The bifunctional cascade catalysis is successfully demonstrated in microfluidics for the real-time colorimetric detection of glucose with a low detection limit of 0.8 μM within 30 s. The artificial nanozymes with physiological functions provide the feasible strategies for mimicking the natural enzymes and realizing the biomedical diagnostics with a smart and miniature device.

## Abstract (web summary)

Glucose oxidase (GOx) and horseradish peroxidase (HRP) are used as an enzymatic cascade for blood glucose monitoring. Here, the authors developed a metal-free, graphitic carbon nitride-based nanozyme with GOx- and HRP-like activities and combine it with a microfluidic reactor for real-time glucose monitoring.

## Introduction

Natural enzymes with high substrate specificity and catalytic efficiency prevail to mediate the biological processes in living organisms under mild reaction conditions^1,2^. However, because protein enzymes suffer from high cost of production and intrinsic instability, nanomaterials-derived artificial enzymes, nanozymes, have been extensively investigated to imitate the protein enzymes in biomimetic chemistry^3,4^. For example, glucose oxidase (GOx) and horseradish peroxidase (HRP) as the prototype enzyme pair have been often employed in enzyme cascade catalysis particularly for blood glucose monitoring, and various nanozymes have been developed for their applications to enzymatic reactions. Since the first peroxidase-like nanozymes of magnetite was reported^5^, a series of oxide-^6,7^, metal-^8,9^, and carbon-based^10–13^ nanomaterials with good stability and specificity have been employed to mimic HRP for the peroxidation of 3,3’,5,5’-tetramethylbenzidine (TMB) and 2,2’-azino-bis(3-ethylbenzothiazoline-6-sulphonic acid)-diammonium salt (ABTS) in the presence of hydrogen peroxide (H_2_O_2_) or in cascade glucose detection^14–17^. In such nanozyme systems, H_2_O_2_ is generated from glucose oxidation in the presence of GOx and the resulting H_2_O_2_ is subsequently utilized by nanozymes to oxidize chromogenic substrates through their peroxidase mimicking for colorimetric detection of the glucose level (see Supplementary Table 1). In the development of peroxidase-like nanozymes for cascade glucose detection, the production of intermediate H_2_O_2_ is the main rate-determining step in the bi-enzymatic reaction, and the glucose-GOx system suffers from the poor atom efficiency^18^. Therefore, the efficient H_2_O_2_ production using an alternative GOx-like nanozyme that plays the bifunctional roles (both GOx-like and peroxidase-like) is urgently required. Although there have been some reported examples of bifunctional oxidase-peroxidase mimicking nanozymes, all of them are based on expensive noble metal catalysts (see Supplementary Table 2). From this point of view, the metal-free bifunctional nanozymes consisted of earth-abundant elements only are highly desired.

Compared with the common methods of H_2_O_2_ production such as anthraquinone method^19^ and noble metal-based catalysis^20^, the photocatalytic generation of H_2_O_2_ through the proton-coupled electron transfer to dioxygen (eq. 1) is highly desirable since it does not need H_2_ gas reagent and the process operating in ambient condition is eco-friendly^21,22^. The major challenges in the photocatalytic production of H_2_O_2_ are to enhance the selectivity of two electron transfer to dioxygen and to minimize the decomposition of in situ produced H_2_O_2_^23,24^. The graphitic carbon nitride (*g*-C_3_N_4_: GCN) is an ideal material that can hinder the in situ decomposition of H_2_O_2_ since it has lower adsorption for H_2_O_2_. In addition, chemical functional groups and electronic properties of the GCN can be easily varied through simple modification^25–27^, which makes it a promising photocatalyst for H_2_O_2_ production. Although GCN with good biocompatibility has been also employed as a peroxidase-mimicking nanozyme for glucose detection^28–30^ (Supplementary Table 1), its GOx-mimicking behaviour has never been explored in enzymatic tandem cascade (domino) reactions for colorimetric detection of glucose.

1 $${\mathrm{O}}_{\mathrm{2}}{\mathrm{ + 2H}}^{\mathrm{ + }}{\mathrm{ + 2e}}^{\mathrm{-}} \to {\mathrm{H}}_{\mathrm{2}}{\mathrm{O}}_{\mathrm{2}}{\mathrm{;E}}^ \circ {\mathrm{ = 0}}{\mathrm{.695V}}_{{\mathrm{NHE}}}{\mathrm{.}}$$

Herein, we propose the example of a bifunctional metal-free nanozyme of modified GCN, which performs the dual roles for oxidase-mimicking in glucose oxidation and peroxidase-mimicking in chromogenic substrate oxidation under irradiation and dark condition, respectively. The selectivity for dioxygen reduction and efficient charge separation promote the in situ photogeneration of H_2_O_2_ from glucose oxidation under visible light. The in situ produced H_2_O_2_ is then utilized for subsequent peroxidation of a chromogenic substrate on the same modified GCN to complete the bifunctional oxidase-peroxidase mimicking in glucose detection. Finally, the GCN-based bifunctional enzyme-mimicking cascade catalysis is successfully demonstrated in a continuous flow microfluidic reactor for rapid and sensitive real-time monitor of glucose.

## Results

### Design and characterization of the bifunctional nanozyme

**Fig. 1:**
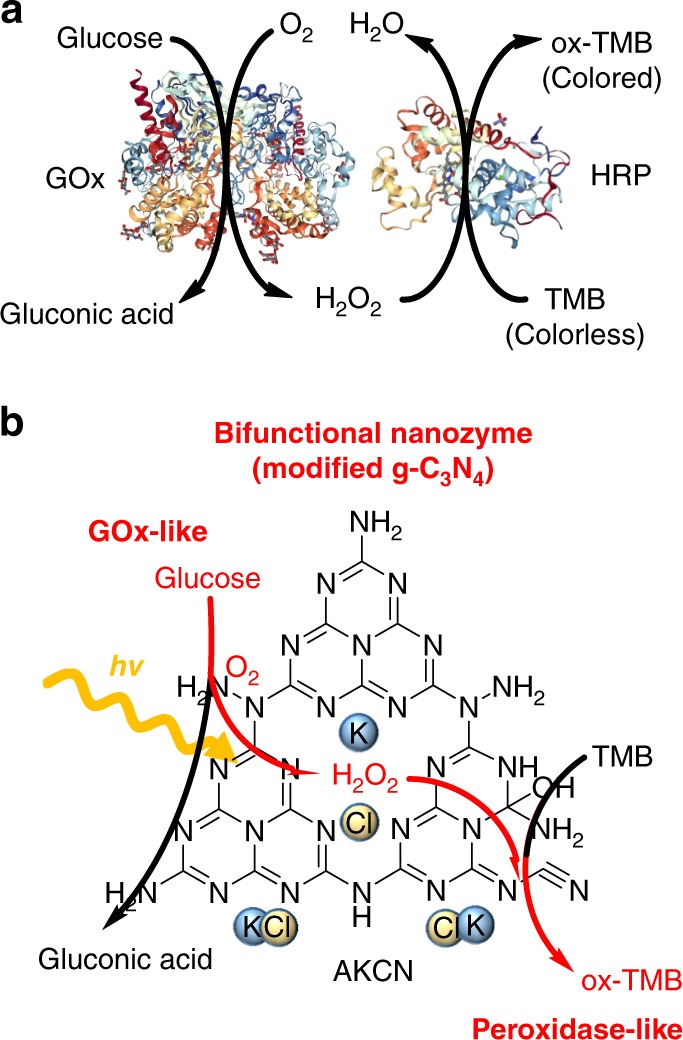
Comparisons of glucose detection model in cascade reaction systems. **a** Glucose detection using enzymes: colorimetric detection of glucose using glucose oxidase (GOx) and horseradish peroxidase (HRP). **b** Glucose detection using a synthetic bifunctional nanozyme: photocatalytic aerobic oxidation of glucose with in situ production of H_2_O_2_ on AKCN (modified GCN). In situ H_2_O_2_ generated from glucose oxidation is subsequently supplied as a fresh reactant to mimic the peroxidase function as shown in **a**. Source data are provided as a Source Data file

When coupled with the glucose-GOx, the in situ production of H_2_O_2_ from glucose oxidation serves for the subsequent HRP-mediated peroxidation of TMB for colorimetric detection of glucose (Fig. 1a)^15,31^. In this work, we employed the modified GCN as an artificial enzyme (nanozyme) that mimics the dual roles of GOx (oxidizing glucose with in situ production of H_2_O_2_) and HRP (TMB oxidation using in situ generated H_2_O_2_) in natural enzyme system (Fig. 1b). The modified GCN functions as a GOx-like photoenzyme that photocatalytically oxidizes glucose with the concurrent reduction of O_2_ to H_2_O_2_ under visible light. The in situ generated H_2_O_2_ is then reductively decomposed (eq. 2 and 3)^10^ with oxidizing TMB on the modified GCN, which mimics HRP in the dark condition.

2 $${\mathrm{H}}_{\mathrm{2}}{\mathrm{O}}_{\mathrm{2}}{\mathrm{ + H}}^{\mathrm{ + }}{\mathrm{ + e}}^{\mathrm{-}} \to {\mathrm{OH}}^ \bullet {\mathrm{ + H}}_{\mathrm{2}}{\mathrm{O,E}}^ \circ {\mathrm{ = 1}}{\mathrm{.14V}}$$

3 $${\mathrm{H}}_{\mathrm{2}}{\mathrm{O}}_{\mathrm{2}}{\mathrm{ + 2H}}^{\mathrm{ + }}{\mathrm{ + 2e}}^{\mathrm{-}} \to {\mathrm{2H}}_{\mathrm{2}}{\mathrm{O,E}}^ \circ {\mathrm{ = 1}}{\mathrm{.763V}}$$

**Fig. 2:**
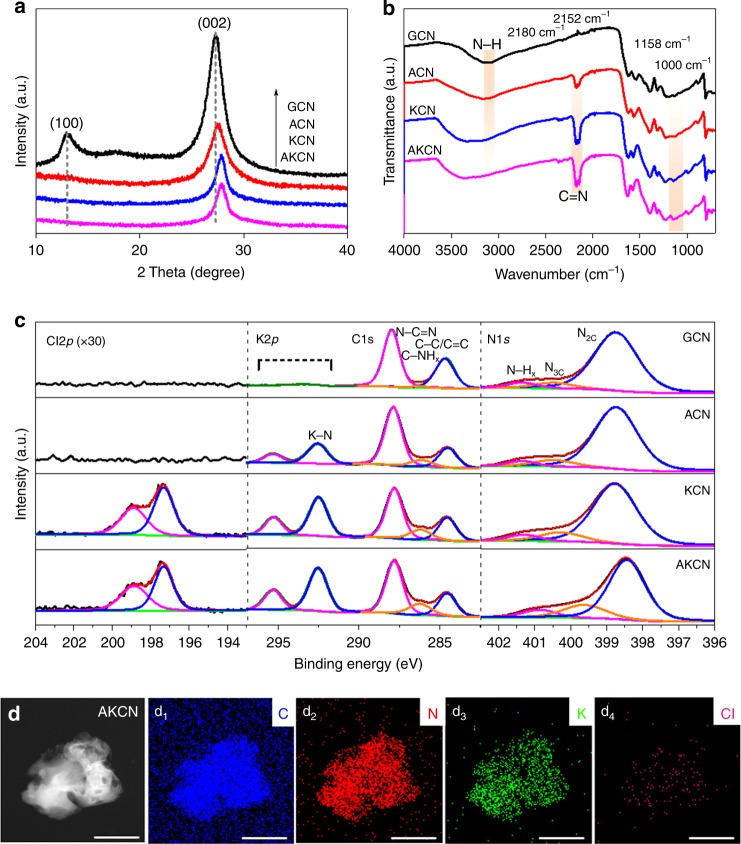
Characterization of the bifunctional nanozyme. **a** XRD patterns, **b** FTIR spectra, and **c** Cl 2*p*, K 2*p*, C 1*s* and N 1*s* XPS spectra of GCN, ACN, KCN, and AKCN. The red line and green line represent the peak simulation and baseline. The deconvoluted peaks was identified from the pink, blue, and orange line. **d** TEM image of AKCN with corresponding elemental mapping (**d**_**1**_–**d**_**4**_). The scale bar is 1 µm in figures. The colours of blue, red, green, and pink represent the elemental components of **d**_**1**_ C, **d**_**2**_ N, **d**_**3**_ K, and **d**_**4**_ Cl, respectively. Source data are provided as a Source Data file

The GCN photocatalyst is ideally suited for the visible light-induced synthesis of H_2_O_2_ since the generated H_2_O_2_ has little adsorption ability onto GCN surface and its in situ photodecomposition can be minimized. The pure GCN, however, has a low photoactivity for H_2_O_2_ production and therefore the modification of GCN with multiple elements doping has been tried to enhance the photoefficiency significantly^34,35^. A recent study that synthesized KPF_6_-modified GCN achieved an apparent quantum yield of 24% at 420 nm for the production of H_2_O_2_ in ethanol solution^35^. To further increase the photoefficiency of H_2_O_2_ production to mimic the high efficiency of GOx enzyme, we developed a modified GCN. The synthesis of pristine GCN via the one-pot thermal polycondensation of melamine^36^ was modified by introducing KOH or/and KCl. The modified GCN samples incorporated with KOH, KCl, and both are referred as ACN, KCN, and AKCN, respectively. The resulting AKCN exhibited the main phase of GCN in the XRD spectrum (Fig. 2a). The fact that the (002) peak shifted along with the disappearance of (100) peak indicates the presence of K interaction among the interlayer and in-plane^36–38^, which contributed to the formation of tightly stacked layers on modified GCN compared with pristine GCN, judging from the FESEM images (Supplementary Figure 1). In FTIR spectra, the surface hydroxyl group grafting (–C–OH) over AKCN was evidenced from the appearance of extra bands at 1000, 1158, and 2152 cm^−^^1^ (Fig. 2b), which indicates the replacement of terminal –NH_2_ groups by hydroxyl groups after KCl and KOH introduction^32,34^. In addition, the noticeable appearance of the new band around 2180 cm^−^^1^ in ACN, KCN, and AKCN samples can be ascribed to the cyano groups (–C≡N) transformed from the terminal –C–NH_2_ at the melon structural unit^33,36^.

The compositions and chemical states in pristine and modified GCN samples were further analysed through XPS survey analysis (Supplementary Figure 2). In the high resolution C 1*s* spectra of pristine GCN (Fig. 2c), the typical components around 288.2, 286.4, and 284.8 eV can be indexed as N–C = N, C–NH_x_, and adventitious carbon, respectively. The markedly enhanced peak around 286.4 eV in ACN, KCN, and AKCN indicates the presence of more C–NH_x_ groups, which subsequently induces more tricoodinated N_3C_ species (around 400.0 eV) deriving from the cyano group formation^38^. It should be noted that most peaks of N 1*s* and C 1*s* in AKCN exhibit a clear shift to lower binding energies compared with pristine GCN (Fig. 2c), which reveals the effect of KCl and KOH modification on the chemical bonding in the GCN structure. The binding energies of K 2*p* (292.5 eV and 295.2 eV) and Cl 2*p* (197.3 eV and 198.9 eV) peaks are also significantly shifted from those of KCl (K 2*p*_3/2_: 293.6 eV; Cl 2*p*: 199.2 eV), which supports that K^+^ and Cl^−^ ions interact with the surrounding C and N atoms^37^. The distribution of K and Cl elements incorporated within AKCN structure is clearly seen from EDS mapping along with the backbone elements of C and N (Fig. 2d). A small O 1*s* peak was observed in the survey scan (Supplementary Figure 2a) due to the adventitious oxygen-containing species (–C–OH) grafted on the surface, which is consistent with the FTIR result. In comparison with other counterparts, AKCN with higher XPS elemental concentrations of K (8.91 at%) and Cl (0.63 at%) that electronically interact with the chemical structure of GCN should influence the migration and separation of electrons and holes. All the above analysis revealed that AKCN successfully incorporated K and Cl atoms in the GCN framework to bridge the interlayers for efficient charge separation.

### Photocatalytic H_2_O_2_ production

**Fig. 3:**
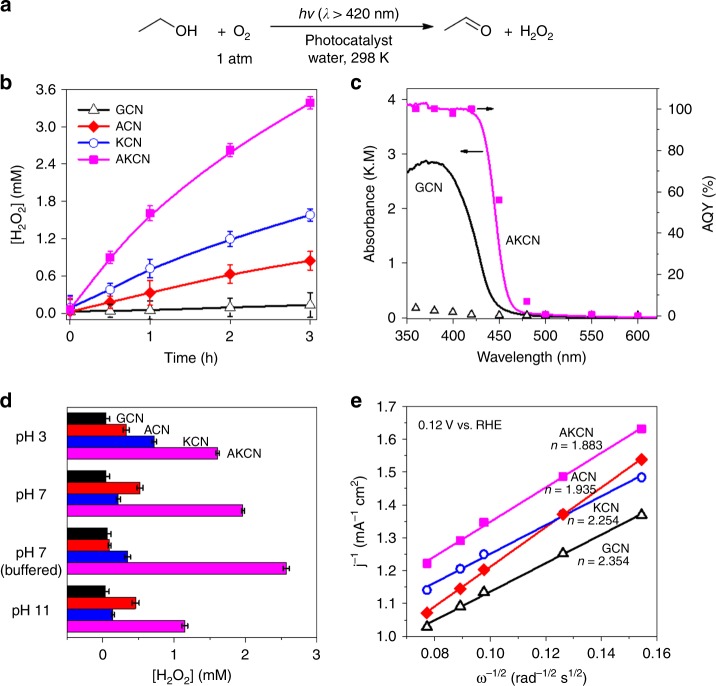
Photocatalytic production of H_2_O_2_ and the properties of modified GCN. a Photocatalytic reaction scheme of aerobic oxidation of alcohol coupled with H_2_O_2_ production. Experimental conditions: photocatalyst (0.5 g L^−^^1^) with 10 vol% EtOH under visible light illumination (*λ* ≥ 420 nm), T = 25°C, water or phosphate buffer (0.1 M, pH 7). **b** Time profiles of H_2_O_2_ photoproduction at pH = 3. **c** Apparent quantum yield (AQY) of H_2_O_2_ production as a function of irradiation wavelength (0.1 M phosphate buffer, pH 7). The black open-triangle and pink solid-square represent the AQY of GCN and AKCN, respectively. **d** pH effect on the photocatalytic production of H_2_O_2_ over pure and modified GCN in 1 h irradiation. **e** The Koutecky–Levich plots obtained via RDE measurements in KOH (pH 13) solution with continuous O_2_ purging at 0.12 V (vs. RHE). The error bar represents the standard deviation from the repeated experiment after three times. Source data are provided as a Source Data file

The ideal atom efficiency of sunlight-driven H_2_O_2_ generation (overall reaction as eq. 4) can be up to 100% by coupling the efficient water oxidation (eq. 5) and the selective two-electron reduction of O_2_ (eq. 1). However, the photocatalytic production of H_2_O_2_ has been commonly investigated by utilizing alcohols as an electron donor since the water oxidation (eq. 5) is inefficient^21,22^. This study aims to utilize glucose as an electron donor instead of alcohols and employed GCN as a photoenzyme that mimics the role of GOx under visible light. Since the activity of pure GCN for the production of H_2_O_2_ is low, the pure GCN was further modified by incorporating KOH, KCl, and both (KOH/KCl). ACN and KCN were optimized for the content of KOH and KCl and characterized by XRD and FTIR (Supplementary Figure 3 and 4). The photocatalytic production of H_2_O_2_ from the resultant samples was tested in the presence of ethanol (see Fig. 3a), which should serve as a proxy test for H_2_O_2_ production coupled with glucose oxidation^25^. The highly selective H_2_O_2_ formation under visible light was enabled by promoting two-electron reduction of O_2_ via the rapid formation of 1,4-endoperoxide species in the polymeric GCN structure^23^. The in situ generated H_2_O_2_ can be also utilized as a Fenton reagent to accelerate the photocatalytic oxidation process^34,38^. Among various GCN samples modified with different reagents containing alkali metal ions and halide ions, the GCN incorporated with KCl and KOH exhibited markedly higher activities (Supplementary Figure 3d and 4d). To investigate whether the optimal molar ratio can be different when both KOH and KCl are copresent, we carried out an additional optimization of KOH content in the presence of a fixed content of KCl (Supplementary Figure 5). By comparing Supplementary Figure 3 and 5, we found that the optimal content of KOH was not influenced by the presence of KCl. Figure 3b shows that AKCN with global optimization of KOH and KCl produced the highest amount of H_2_O_2_ (3.4 mM) in 3 h, which was 2, 4, and 24 times higher than KCN (1.58 mM), ACN (0.85 mM), and pure GCN (0.14 mM), respectively. In this case, the effect of catalyst surface area on the photoactivity seems to be insignificant because AKCN with the highest activity had the smallest S_BET_ (Table 1). As for the wavelength-dependent activity, Fig. 3c shows that the photoactivity of AKCN well matches the absorption spectral profile whereas the photoactivity of pure GCN is negligible despite its visible light absorption in the same wavelength range. In particular, AKCN exhibited the apparent quantum yield (AQY) close to 100% in the range of 320–420 nm and 56% at 450 nm, which seems to be the highest reported AQY of H_2_O_2_ production in the visible light range to our knowledge.

4 $${\mathrm{2H}}_{\mathrm{2}}{\mathrm{O + O}}_{\mathrm{2}} \to {\mathrm{2H}}_{\mathrm{2}}{\mathrm{O}}_{\mathrm{2}}$$

5 $${\mathrm{2H}}_{\mathrm{2}}{\mathrm{O + 4h}}^{\mathrm{ + }} \to {\mathrm{O}}_{\mathrm{2}}+{\mathrm{4H}}^{\mathrm{ + }}$$

**Table 1:** Structural characteristics of various carbon nitride samples prepared at 550 ^o^C

Samples	Reagent molar ratio	S_BET_ (m^2^ g^−^^1^)	V_p_ (cm^3 ^g^−1^)	E_g_ (eV)
GCN	Melamine	7.6	0.05	2.79
ACN	Melamine + KOH/(1:0.002)	4.8	0.03	2.77
KCN	Melamine + KCl/(1:0.08)	3.0	0.02	2.74
AKCN	Melamine + KOH + KCl /(1:0.002:0.08)	2.2	0.01	2.70

It is noted that all the modified GCN (ACN, KCN, and AKCN) exhibited more negative zeta potentials in comparison with pure GCN (Supplementary Figure 6). The higher negative surface charge on the modified GCN should be favourable for H_2_O_2_ production (eq. 1) since the supply of protons onto the negatively charged catalyst surface should be facilitated by the electrostatic attraction^24,39^. AKCN produced the highest amount of H_2_O_2_ consistently in a wide pH range (pH 3–11) than GCN, ACN, and KCN. When comparing different photocatalysts, the activities were measured at pH 3 because the activity differences among different photocatalysts were most clearly observed at this pH (see Fig. 3d). However, it should be noted that the optimal catalyst composition determined at pH 3 was the same as that determined at phosphate buffer condition (see Supplementary Figure 3c and 4c). In this case, AKCN exhibited the highest enhancement at neutral phosphate buffer solution as a result of the possible specific phosphate promotion (Fig. 3d and Supplementary Figure 5)^40,41^, which concurrently matches the optimal condition of GOx action. The production of H_2_O_2_ in the phosphate buffer solution increased in the order of (AKCN > KCN > ACN > GCN), which is the same trend as observed at pH 3, and gradually reached the photostationary state in 16 h irradiation (Supplementary Figure 7). This indicates that the formation and decomposition rate of H_2_O_2_ is balanced after a prolonged irradiation^42,43^. In addition, all catalysts (GCN, ACN, KCN, AKCN) exhibited significantly hindered activity for the decomposition of H_2_O_2_ under visible light (see Supplementary Figure 7, left axis). Unlike metal oxide photocatalysts with high adsorption for in situ generated H_2_O_2_, GCN catalysts with little adsorption ability for H_2_O_2_ have insignificant activity for H_2_O_2_ decomposition and are ideally suitable for the production of H_2_O_2_^39,44^. From the viewpoint of stability, AKCN exhibited a good stability without loss of activity during repeated cycles (Supplementary Figure 8). On the other hand, the characteristics of the electron transfer to O_2_ on AKCN was investigated by the rotating disk electrode (RDE) analysis, that enables the estimation of the number of electrons (n) transferred to O_2_ from the slope value of Koutecky–Levich plots (Fig. 3e). The estimated “n” values were close to 2 for pure GCN, ACN, KCN, and AKCN, which indicates that dioxygen molecules is selectively reduced by two-electron transfer only^23,25^.

### Improvement of charge separation

As for the light absorption, the absorption edge of KCN, ACN, and AKCN is progressively redshifted with respect to pristine GCN (Supplementary Figure 9a), which corresponds to the bandgap change from 2.79 eV to 2.70 eV (Table 1) according to the Tauc plot anlalysis (Supplementary Figure 9a inset). The valence band position could be determined from the valence band XPS (Supplementary Figure 9b). This combined with the above bandgap determination shows that conduction band (CB) and valence band (VB) levels slightly shifted to the positive potential after the modifications of KCl and KOH (Supplementary Figure 9c). However, such a small potential shift cannot provide enough overpotentials for raising the photocatalytic activity of AKCN to the level that is significantly higher than that of GCN, ACN, and KCN. This implies that the markedly high photoactivity of AKCN might be related to not only the thermodynamic factors, but also the kinetic factors, which should facilitate the charge separation and transfer in the modified structure of GCN. How the photocatalytic activity is strongly enhanced for H_2_O_2_ production in AKCN is discussed in relation with the calculated electronic properties in a later section.

**Fig. 4:**
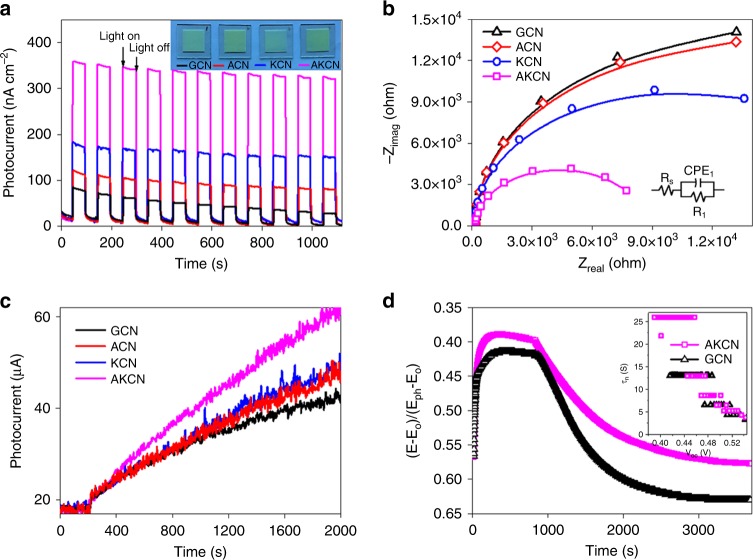
Photoelectrochemical behaviours of pure and modified GCN. **a** Transient photocurrent responses (inset: images of different electrodes). **b** Electrochemical impedance spectra (EIS). In the simulated electrical equivalent-circuit model (inset), R_S_, R_1_, and CPE represent as solution resistance, charge transfer resistance, and double layer capacitance, respectively. **c** Time profiles of Fe^3+/2+^-redox shuttle-mediated photocurrent collected on a Pt electrode in the catalyst suspension. **d** Open-circuit voltage decay (OCVD) measurement (inset: average lifetimes of the photogenerated carriers as a function of the V_oc_). The black open-triangle and pink open-square represent the GCN and AKCN, respectively. Source data are provided as a Source Data file

To characterize the photoelectrochemcial properties, the chopped photocurrent response and electrochemical impedance spectra measurements were further carried out by using the as-prepared electrodes (Fig. 4a inset). The synergetic effect of K incorporation and surface alkalization on charge separation was clearly demonstrated from the observation that AKCN exhibited the highest photocurrent among all tested electrodes (Fig. 4a). It has been reported that the surface alkalization improved the charge separation on ACN compared to GCN^45^ and the K-incorporation bridged structure facilitated the anisotropic electron flow and separation^37,46^. When K incorporation was coupled with surface alkalization, AKCN exhibited the highest photocurrent, which was consistent with its smallest arc radius in Nyquist plot analysis (Fig. 4b). The highly enhanced interfacial charge transfer on AKCN was further confirmed by measuring the Fe^3+/2+^ shuttle-mediated photocurrent in the catalyst suspension under visible light, which was also highest with AKCN (Fig. 4c). In the transient open-circuit voltage decay (OCVD) measurements, AKCN exhibited higher open-circuit voltage and slower photovoltage decay than pure GCN (Fig. 4d), which indicates that the charge recombination in AKCN is hindered for prolonging the lifetimes of charge carriers (Fig. 4d inset)^47^.

### Glucose oxidase-like activity

**Fig. 5:**
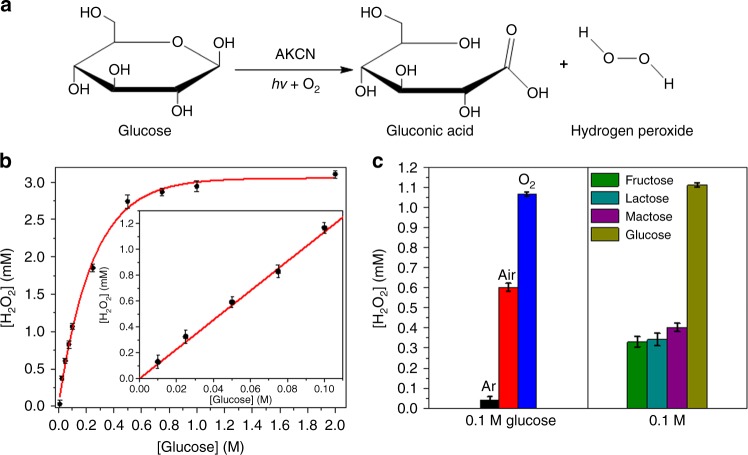
Photocatalytic aerobic oxidation of glucose with the concurrent production of H_2_O_2_. **a** Scheme of GOx-like reaction of AKCN (photoenzyme). **b** H_2_O_2_ production as a function of glucose concentration (inset: enlarged plot in the linear region) in the phosphate buffer (0.1 M, pH 7) suspension of photoenzyme (0.5 g L^−1^) under visible light illumination (*λ* ≥ 420 nm), T = 25 ^o^C. **c** H_2_O_2_ generated by AKCN photoenzyme in the presence of different kinds of saturated gas (0.1 M glucose) and carbohydrate substrate (0.1 M). The error bar represents the standard deviation from the repeated experiment after three times. Source data are provided as a Source Data file

AKCN exhibited the superior photoactivity for H_2_O_2_ generation in neutral phosphate buffer solution (0.1 M, pH 7), which happened to be coincident with the typical working condition of glucose oxidase (GOx). When alcohol is replaced by glucose as the electron and proton donor, the GOx-like activity of AKCN can be induced under visible light to produce H_2_O_2_ and gluconic acid from the glucose oxidation (see Fig. 5a). The photocatalytic GOx-mimicking behaviours of AKCN were successfully demonstrated by in situ photoproduction of H_2_O_2_ (Fig. 5b), which was proportional to [glucose]: the production of H_2_O_2_ exhibited an excellent linear correlation with [glucose] up to 0.1 M (Fig. 5b inset). The production of CO_2_ from the photocatalytic oxidation of glucose was negligibly small (2 µmol), compared with that of H_2_O_2_ (0.8 mM, in the O_2_-saturated glucose buffer solution (1 M) after 1 h irradiation). This indicates that the photocatalytic mineralization of glucose is prohibited in the present condition and glucose is selectively phototransformed to gluconic acid on AKCN as illustrated in Fig. 5a. The concentration of H_2_O_2_ was determined by the colorimetric N,N-diethyl-1,4-phenylene-diamine sulfate (DPD) method (DPD oxidized by H_2_O_2_ and POD), which exhibited a good linearity up to 1.5 mM H_2_O_2_ (Supplementary Figure 10). The photocatalytic production of H_2_O_2_ on AKCN was negligibly small in Ar-saturated condition (O_2_-free) and progressively higher in air-saturated and O_2_-saturated condition (Fig. 5c), which supports that H_2_O_2_ production is derived mainly from the selective two-electron reduction of O_2_ when coupled with the photooxidation of glucose. In accord with its higher AQY in EtOH, AKCN also exhibited highest AQY (close to 100%) for H_2_O_2_ production in glucose buffer solution under the visible light (Supplementary Figure 11). Similar to the behaviour of GOx with good specificity for glucose, this photoenzyme of AKCN also exhibited the higher selectivity for glucose oxidation when compared to other glucose analogues, such as fructose, lactose, and maltose (Fig. 5c). Overall, AKCN seems to mimic the behaviour of GOx (producing H_2_O_2_ along with the simultaneous oxidation of glucose) under visible light irradiation. The resulting H_2_O_2_ can be furthered to combine with peroxidase (HRP) to oxidize chromogenic substrates for colorimetric detection of glucose.

### Peroxidase-like activity

**Fig. 6:**
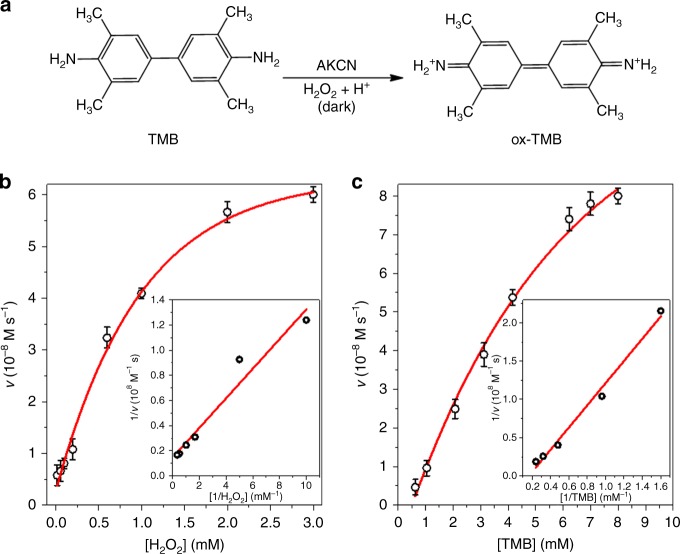
Steady-state kinetics for the peroxidase mimicking dark-reaction of AKCN. **a** Scheme of the peroxidase-like reaction. The reaction rate as a function of **b** [H_2_O_2_] and **c** [TMB] in the acetate buffer (0.1 M, pH 4) suspension of AKCN (0.5 g L^−^^1^) with TMB and H_2_O_2_ for 10 min incubation. Inset: Lineweaver–Burk plots. The error bar represents the standard deviation from the repeated experiment after three times. Source data are provided as a Source Data file

On the other hand, the intrinsic peroxidase-like activity of AKCN was also tested in the AKCN-TMB-H_2_O_2_ system, where the chromogenic substrate of TMB was oxidized under the dark condition (Fig. 6a). The resulting ox-TMB generated deep blue colour (Supplementary Figure 12). This demonstrated the bifunctional biomimetic roles of AKCN: (1) AKCN plays the role of photoenzyme to mimic GOx that oxidizes glucose with the concurrent production of H_2_O_2_ under visible light; (2) AKCN mimics HRP that oxidizes the chromogenic TMB to induce blue coloration in the dark. The peroxidase-mimicking activities of AKCN were compared with those of HRP with varying pH and temperature (Supplementary Figure 13). The operating pH and temperature ranges are quite similar between AKCN and HRP, but AKCN with graphitic structure exhibited consistently higher activities than HRP above 30 °C.

The peroxidase mimicking activities of AKCN were systematically investigated by varying one substrate concentration while keeping the other one constant. It was found that the steady-state kinetics well followed the typical Michaelis–Menten model in the tested concentration range of H_2_O_2_ (Fig. 6b) and TMB (Fig. 6c). From the Lineweaver–Burk double reciprocal plots (inset), the corresponding kinetic parameters of maximum initial velocity (*V*_max_) and Michaelis–Menten constant (*K*_m_) were obtained from the slopes and intercepts of the fitted lines, which are summarized in Supplementary Table 3. Compared to ferric oxide (154) and HRP (3.7)^5^, the lower *K*_m_ value of AKCN (0.79) for substrate H_2_O_2_ represents its higher binding affinity for H_2_O_2_^48^, indicating the lower concentration of H_2_O_2_ required to reach the maximal activity of *V*_max_. In contrast, the *K*_m_ value of AKCN with respect to TMB was significantly higher than that of HRP, consistent with the higher TMB concentration required to achieve the maximal activity through the mediation of charge transfer between TMB oxidation and H_2_O_2_ reduction^49^. Same as the case of electrons transfer from graphene to H_2_O_2_^10^, the AKCN with graphene-like structure can also facilitate the electron transfer from TMB (TMB^Ox^/TMB 1.13 V) to H_2_O_2_ (H_2_O_2_/H_2_O 1.776 V) for intrinsic peroxidase-like activity^50,51^. However, the detailed mechanism of this process on AKCN is unclear and needs to be studied further. It is interesting to note that the peroxidase-like activity of AKCN is specific to TMB only, not effective to ABTS that is a common substrate for peroxidase enzymes (Supplementary Figure 14). This might be ascribed to the electrostatic repulsion between the negatively charged AKCN (see Supplementary Figure 6) and anionic ABTS. The peroxidase mimicking activity of AKCN specific to TMB indicates that the modified structure of AKCN facilitates the selective electron transfer from TMB to H_2_O_2_. Inspired by the higher affinity of AKCN to H_2_O_2_ in peroxidase-like activity, a colorimetric H_2_O_2_ detection with a low detection limit of 0.015 mM was performed (Supplementary Figure 15). As demonstrated above, AKCN possesses both GOx-mimicking and HRP-mimicking behaviuors. This motived us to perform the colorimetric detection of glucose in cascade reactions (sequential combination of both behaviours) through the in situ H_2_O_2_ production from AKCN-catalysed glucose oxidation.

### Enzyme-like cascade reactions in batch and microfluidic modes

**Fig. 7:**
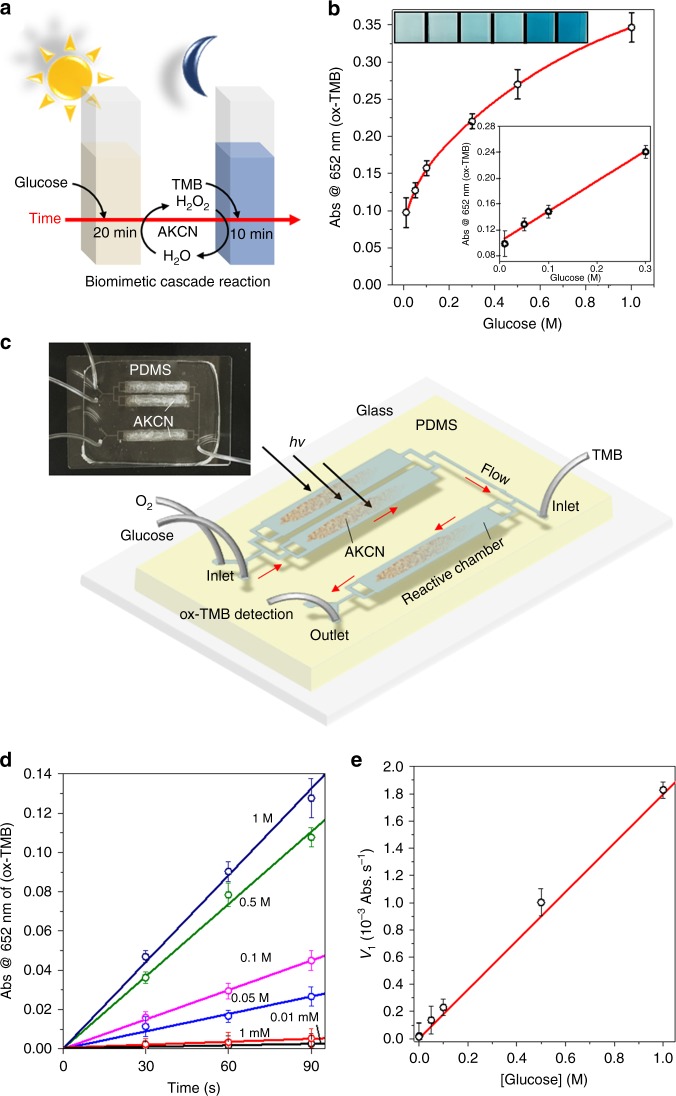
Comparative enzymatic cascade reaction for glucose detection. **a** Scheme of the cascade reaction with continuous O_2_-purging in a batch mode. **b** The concentration-response curve with the linear calibration plots (inset) and colour change (inset) for glucose detection in batch reactor. The AKCN was employed as an oxidase (0.1 M phosphate buffer, pH 7) and a peroxidase (0.1 M acetate buffer pH 4), sequentially in cascade reaction. **c** Scheme of the cascade reaction in a microfluidic device and actual device image (inset). **d** TMB oxidation by AKCN in the microfluidic channel (monitored as a function of time) for glucose detection in the concentration range of 0.01 mM–1.0 M. **e** The linear calibration for initial TMB oxidation rate (*v*_i_) vs. glucose concentration. The error bar represents the standard deviation from the repeated experiment after three times. Source data are provided as a Source Data file

Based on the above GOx- and peroxidase-like activities, the artificial enzymatic cascade reaction carried out by the bifunctional AKCN, instead of GOx-HRP bi-enzymatic reaction, was comparatively tested for glucose detection in a batch and a microfluidic reactor as illustrated in Fig. 7a, c. In the batch reactor with continuous O_2_ purging, H_2_O_2_ was generated through the photocatalysis of AKCN/glucose for 20 min under visible light irradiation (*λ* ≥ 420 nm). The in situ generated H_2_O_2_ in the photo-stage was then consumed in the following dark-stage where TMB is oxidized by H_2_O_2_ on AKCN. The correlation between [glucose] and [H_2_O_2_] generation after the photo-stage in the reactor was successfully confirmed (Supplementary Figure 16). After the TMB injection, the in situ generated H_2_O_2_ was subsequently depleted by the peroxidase-mimicking action of AKCN with simultaneous appearance of blue colour (images inset, indicating the production of ox-TMB)^52^. Similar to the GOx-peroxidase-coupled enzymatic system, the cascade enzymatic mimicking was successfully achieved in the combined system of (1) AKCN/glucose/*hv* with in situ production of H_2_O_2_ and (2) AKCN/H_2_O_2_/TMB (dark). As a result, the production of ox-TMB (monitored by absorption at 652 nm) gradually increased with the glucose concentration ranged from 0.01 M to 1 M (Fig. 7b), and a good linearity was established in the range of 0.01 M < [glucose] < 0.3 M (Fig. 7b inset), which was sensitive enough (with a limit detection of 0.07 mM) to distinguish between the healthy (3–8 mM) and diabetic body (9–40 mM) by monitoring the blood glucose level^50,53^.

The above enzyme-mimicking cascade reaction can be more facilitated with enhanced mass transfer in a continuous flow microfluidic reactor, which enables to miniaturize the system as a portable real-time monitoring platform^54^. For this purpose, a feasible microfluidic device for glucose detection in a small volume was fabricated by stacking a PDMS substrate with AKCN-coated flow channels (5 mm width, 0.044 mm height, 30 mm length) onto a slide glass (Fig. 7c and inset, see experimental method and Supplementary Figure 17). The cascade reaction was conducted along two flow channels that are serially connected with 30-mm-long channel; the former has the AKCN-coated channel under visible light irradiation (GOx-mimicking part) and the latter mixes the in situ generated H_2_O_2_ (from the former) with TMB injection (HRP-mimicking part). In other words, the mixed glucose-O_2_ from the flow vessel was transferred into the flow channel by a syringe-pump, where the AKCN (0.25 mg per chamber) immobilized on the channel wall catalyses the H_2_O_2_ generation under visible light irradiation. The outlet flow from the first channel was continuously fed into the second flow channel (shielded from the irradiation) for the subsequent peroxidase-mimicking reaction on AKCN. TMB was introduced through a separate inlet in the head of the second flow channel. The solution in the second flow channel was immediately turned blue (Supplementary Figure 17e) due to the formation of ox-TMB and the resulting outflow out of the channel can be analysed for its absorbance at 652 nm in real time. Since H_2_O_2_ was equilibrate proportion to the oxidized glucose (one molar molecule of glucose converted to one molar H_2_O_2_) in GOx mimicking, the further continuously catalysis of quantitative [TMB] with accurate [H_2_O_2_] (detected from DPD method) might offer a promising indicator in glucose assay originated from the calibration curve of [glucose] against the TMB and H_2_O_2_ (Supplementary Figure 18).

The initial reaction rate (*v*_i_) can be calculated from the plot of the ox-TMB absorbance as a function of time (Fig. 7d). The *v*_i _was linearly correlated with [glucose] (Fig. 7e), which enables the quantification of the glucose concentration from the calculated *k*_obs_ (1.7 Abs. s^−^^1^ mM^−1^). It is worth noting that this analytical method based on the photonic microfluidic cascade reaction can be used to detect the unknown glucose concentration from the calibration curve within 30 s by measuring *v*_i_ (Abs *=* *ɛcl*, *ɛ* = 39000 L·mol^−^^1^·cm^−1^ for TMB). It exhibited the limit of detection around 0.8 μM in microfluidic device, which is sensitive enough for practical application since the clinical glucose concentration is greater than 1 mM. The microfluidic biomimetic catalysis exhibited the superior turnover frequency (9.1 h^−^^1^) of H_2_O_2_ generation as 5.3 times high as that of the batch process (1.7 h^−1^), which can be ascribed to the intrinsic characteristics of the microfluidic reactors such as efficient mass transfer and high surface-to-volume ratio (see detailed discussion in Supplementary Note). While all the previously reported bifunctional nanozymes required hours for glucose detection (Supplementary Table 2), the present AKCN-based nanozyme employed in a microfluidic device exhibited a much faster cascade catalysis (~30 s) and a lower detection limit (0.8 μM). In addition, the detection limit of the microfluidic device is orders of magnitude lower than that of the batch reactor. The merits of the present nanozyme–microfluidic sensor are highly desirable for point-of-care diagnosis. Overall, this study successfully demonstrated the performance of the microfluidic reactor for biomimetic cascade catalysis reaction as a miniaturized tool for rapid and facile real-time monitor of glucose.

## Discussion

**Fig. 8:**
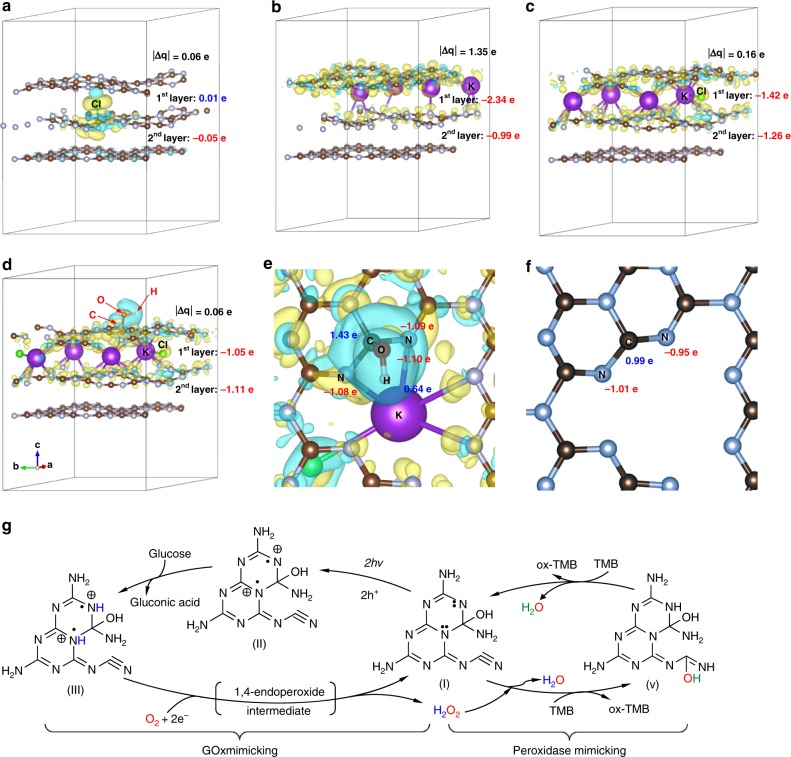
Charge distribution analysis from density functional theory (DFT) calculations. Charge distribution of **a** Cl-GCN, **b** K-GCN, **c** KCl-GCN, **d** KCl-OH-GCN (i.e. AKCN), **e** an enlarged top view of KCl-OH-GCN, and **f** that of pristine GCN. **g** The effect of charge redistribution on the promotion of photocatalytic H_2_O_2_ generation between GCN and AKCN. ǀ∆qǀ represents the absolute value of the difference of the electron distribution between the first and second layer. Yellow colour represents electron accumulation and blue colour represents electron depletion. A negative value means that accumulation of the electron based on the valence electron. Isovalue is taken as 0.002. Brown and grey colour represent carbon and nitrogen, respectively. Source data are provided as a Source Data file

The successful performance of AKCN that achieved the ideal efficiency of about 100% AQY implies that the photogenerated charge carriers in AKCN are efficiently separated with directional charge migration and selective reduction of O_2_ to H_2_O_2_. To understand how the modification of GCN with KCl and KOH modified the structure and charge distribution, density functional theory (DFT) calculations were performed. The optimized location of Cl, K, and OH in the carbon nitride structure and the relative energy of each modified GCN structure are shown in Supplementary Figs. 19–22. It is evident that the electron-rich nitride pots in the three-fold N-bridge linking triazine units are liable to capture and confine the alkali cations of K^+^ in the adjacent layers through the ion-dipole interaction^55^. The K-doped GCN structure with weak interlayer bridging made a relatively large number of electrons accumulated on the first layer (–2.34 e of layer charge) than on the second one (–0.99 e of layer charge) (Fig. 8b), whereas the Cl-doping in GCN did not induce such localization of electrons between the layers (Fig. 8a). As a result, the K-GCN exhibited a high value of the charge difference between the adjacent layers (ǀ∆qǀ = 1.35 e) while that on Cl-GCN (ǀ∆qǀ = 0.06 e) is insignificant. The presence of doped K atoms induces the anisotropic electron density distribution, which is preferably accumulated on the first layer but the additional doping of Cl atoms makes the electron distribution more balanced between the layers (Fig. 8c)^37,46,55^. In other words, when both K and Cl are copresent in the carbon nitride structure, the K-induced electron density polarization can be counterbalanced by Cl to lower ǀ∆qǀ (0.16 e) significantly. This implies that the charge transfers between the layers in KCl-doped carbon nitride are more facilitated than those in pristine GCN, which may provide an explanation for its higher photocatalytic activity. When OH groups are additionally introduced in AKCN (noted as KCl-OH-GCN in Fig. 8d), the DFT calculation predicts that the hydroxyl group is preferably bonded to the surface carbon (Supplementary Figure 22) with inducing an outstanding electron depletion region around the OH group (blue colour) on the first layer (see Fig. 8d). This further decreases ǀ∆qǀ from 0.16 e (Figs. 8c) to 0.06 e (Fig. 8d), which should help the facile charge transfer between adjacent layers. It should be noted that the formation of charge delivery channels in AKCN is critically important to extend the π-conjugated system for facilitating the directional carrier migration between the adjacent layers and assisting the interlayer charge separation^37,46^.

The DFT calculation also shows how the presence of OH group changes the charge distribution over different atoms. The enlarged top view of KCl-OH-GCN (Fig. 8e) shows that the surface carbon atom attached to the OH group carries more positive charge (1.43 e) than the corresponding carbon atom in the pristine GCN (0.99 e in Fig. 8f), furthered to be arising for the neighbour N atoms carry more negative charges (–1.09 e and –1.08 e in Fig. 8e) compared with those on the pristine GCN (–0.95 e and –1.01 e in Fig. 8f)^56^. Based on this calculated result, the proposed reaction scheme is illustrated in Fig. 8g. Upon photoexcitation (Fig. 8g–I), electrons and holes surviving from the fast recombination migrate onto the surface of AKCN (Fig. 8g-II). The holes are preferentially trapped at the N sites (electron rich) adjacent to the C–OH group in the melem unit and subsequently abstract two H atoms to form >NH^+^ sites (blue H atoms in Fig. 8g-III) through the glucose oxidation to gluconic acid^57^. On the other hand, a dioxygen molecule reacts with two electrons trapped in the melem unit, which concurrently abstracts two protons from the >NH^+^ sites (protonated through glucose oxidation), and consequently generates H_2_O_2_ as a product through the intermediate of 1,4-endoperoxide. The sequential reactions of holes and electrons regenerate the melem unit. Therefore, the synergistic improvement of spatial charge separation and local polarization between the interlayers and in-plane is critical for efficient H_2_O_2_ production on AKCN.

On the other hand, in the peroxidase mimicking part, the electrophilic carbon atom in the cyano group abstracts an O atom from H_2_O_2_ and subsequently an H atom from TMB (hydroxyl group formation, bottom part of Fig. 8g–v) with generating ox-TMB^58^. The resulting hydroxyl group further abstract an H atom from another TMB to yield a water molecule and another ox-TMB (hydroxyl group depletion, upper part of Fig. 8g–v). The above two ox-TMB can be protonated in acid buffer to form bluish ox-TMBH^+^. Overall, two TMB molecules are oxidized by one molecule of H_2_O_2_ that is in situ photogenerated on bifunctional AKCN, consistently with their higher affinity of H_2_O_2_ than TMB in the peroxidase-mimicking activity. The combined processes (photogeneration of H_2_O_2_ coupled TMB oxidation in the dark) occurring on AKCN successfully complete the enzymatic cascade reaction.

In summary, a metal-free nanozyme based on modified GCN demonstrated bifunctional enzyme-mimicking behaviours, which combined the roles of oxidase (GOx) and peroxidase (HRP) in a sequential light-dark process. With the intrinsic GOx-like mimicking, the in situ generated H_2_O_2_ from photocatalytic oxidation of glucose served for the subsequent peroxidase-mimicking part. The bifunctional AKCN exhibited a near 100% quantum efficiency of H_2_O_2_ generation and enabled the coupled cascade reactions for colorimetric glucose detection. In addition, such biomimicking catalytic process could be highly accelerated in a microfluidic device, which enabled the real-time monitor of H_2_O_2_ and glucose with a detection limit of 0.8 μM in 30 s. Our results not only certify the successful modification but also clarify the importance of charge separation for cascade reaction with combination of theoretical and experimental fundamental insight. This study provides a design strategy for bifunctional nanozyme capable of generating and subsequently utilizing in situ H_2_O_2_ under ambient condition, which can be potentially applied to a variety of eco-friendly and biomimicking processes involving H_2_O_2_.

## Methods

### Materials

All chemicals were purchased from Sigma–Aldrich or Alfa–Aesar with the highest purity and used without further treatment. Melamine, alkalis chloride and potassium halide, 3,3’,5,5 tetramethylbenzidine dihydrochloride (TMB) were bought from Sigma–Aldrich and used as received. Hydrogen peroxide (35 wt %) was bought from Junsei. Milli-Q water was used for all the experiments.

### Photocatalysts preparation

GCN was simply synthesized as following process^36–38^. 1.5 g of melamine was put into porcelain cup with a cap and calcined at 550 °C for 4 h with a ramping rate of 2.2 °C min^-1^. After heating, the resulting product was gently grounded and treated under ultrasonication for 3 h as an aqueous solution (1 g L^−1^). Then the powder was filtered, washed, and dried at 80 °C for further tests. Alkalized GCN (ACN) was synthesized by similar procedure to GCN, but the proper amount (0.3, 1.0, 2.0, 3.0, 4.0 mmol) of potassium hydroxide was mixed with melamine and grounded together before calcination process. For further comparison of the effect from alkaline metals, 2.0 mmol sodium hydroxide or 2.0 mmol barium hydroxide were, respectively, mixed with melamine instead of potassium hydroxide. K-incorporated GCN (KCN) was synthesized by similar procedure to GCN but the proper amount (0.02, 0.04, 0.06, 0.08, 0.1, 0.15, 0.2 mol) of potassium chloride was mixed with melamine and grounded together before calcination. For further comparison of the effect from alkaline metals, 0.08 mol alkalis chloride (ammonium chloride, sodium chloride, barium chloride,) or 0.08 mol potassium halide (potassium fluoride, potassium bromide, potassium iodide, potassium sulfate) were, respectively, mixed with melamine instead of potassium chloride. Optimized GCN (AKCN) was synthesized by similar procedure to GCN, but the proper amount of potassium hydroxide (2.0 mmol) and potassium chloride (0.08 mol) was mixed with melamine and grounded together before calcination process.

### Photocatalysts characterisation

The phase structures of resultant catalysts were characterised by a PANalytical X’Pert diffractometer with an X’Celerator detector using Cu Kα line (1253.6 eV) and ATR-FTIR spectroscopy (Thermo Scientific Nicolet iS50 FTIR/ATR) on a ZnSe crystal. Diffuse reflectance UV/visible absorption spectra (DR-UVS) were obtained by Shimadzu UV-2600 with an integrating sphere attachment. The reference material of BaSO_4_ was used before the measurement. Zeta potentials of the aqueous suspension (10 mM of NaNO_3_) with catalysts were conducted through an electrophoretic light scattering spectrophotometer (ELS 8000, Otsuka) with controllable pH from HClO_4_ and KOH. The surface atomic properties were analysed by X-ray photoelectron spectroscopy (XPS) by Theta Probe AR-XPS System (Thermo Fisher Scientific, U.K.) with an X-ray source using monochromated Al Kα (*hν* = 1486.6 eV) at KBSI Busan centre. The transmission electron micrographs (TEM) and electron energy loss spectra (EELS) mapping were taken by Cs-corrected JEM-2200F microscope (JEOL) at NINT (Pohang, Korea).

### Photogeneration of H_2_O_2_

The aqueous suspension (distilled water or 0.1 M buffer phosphate) containing the catalyst (0.5 g L^-1^) and ethanol (10 vol%) was prepared at pH 3, followed by sonication and O_2_ purging for 30 min. The photocatalytic H_2_O_2_ generation was conducted under visible light irradiation (*λ* ≥ 420 nm) with continuous O_2_-purging. The colorimetric method employing N,N-diethyl-1,4-phenylene-diamine sulfate (DPD, 97%, Aldrich) reagent was used to determine the concentration of H_2_O_2_. The sample aliquots were collected intermittently during the reaction and then mixed with phosphate buffer, DPD solution, and peroxidase (POD, horseradish, Aldrich) under vigorous stirring. The production of H_2_O_2_ was monitored by measuring the absorbance at 551 nm using a UV/visible spectrophotometer (Libra S22, Biochrom). The detailed method is described elsewhere^32,33^. The apparent quantum yield (AQY) was calculated through the equation (eq. 6),

6 $${\varnothing} {{\mathrm{AQE}}} = \frac{{{\mathrm{(Number}}\,{\mathrm{of}}\,{\mathrm{produced}}\,{\mathrm{H}}_{\mathrm{2}}{\mathrm{O}}_{\mathrm{2}}\,{\mathrm{molecules) \times 2}}}}{{{\mathrm{Number}}\,{\mathrm{of}}\,{\mathrm{incident}}\,{\mathrm{photons}}}}{\mathrm{ \times 100}}$$

where the incident wavelength was adjusted by a monochromator (Newport, Oriel 77250) and the light intensities was measured using a low-power detector (Newport, 818-UV).

### Electrochemical analysis

The transient photocurrent and electrochemical impedance spectroscopy were done by the catalysts coated on ITO glass via spin coating. The measurements were conducted on a potentiostat (Gamry, Reference 600) by three electrode system, where Pt wire as counter electrode, Ag/AgCl as reference electrode, and catalyst coated on ITO as working electrode. 0.2 M Na_2_SO_4_ were used as an electrolyte and pH were adjusted to 3 under continuous Ar purging. The slurry photocurrent measurements were carried out in three electrode system, consist of Pt wire, graphite rod, and Ag/AgCl as working, counter and reference electrodes, respectively. Photocatalysts (1 g L^-1^) were suspended in aqueous solution consisting 1 mM NaClO_4_ (electrolyte) and 1 mM Fe^3+^ (electron shuttle) at pH 1.7 with a bias of +0.7 V (vs. Ag/AgCl).

Electrocatalytic oxygen reduction reaction (ORR) was investigated through linear sweep voltammetry (LSV) (using a Gamry Reference 600 potentiostat) in KOH electrolyte (0.1 M) under continuous O_2_ purging. To prepare the catalyst-coated electrode, the catalyst slurry with Nafion (0.5 wt%) was loaded on the surface of glassy carbon disk (815 µg cm^−^^2^). The resultant working electrode was scanned at a rate of 10 mV s^−^^1^ to cathodic direction in LSV. The reference and counter electrodes were Ag/AgCl (in saturated KCl) electrode and platinum wire, respectively. The rotating disk analysis was performed at the speed of 400–1600 rpm during the ORR. The electron transfer number in ORR can be calculated from the slope of Koutecky–Levich plot, which was constructed according to the Koutecky–Levich equation (eq. 7).

7 $$\frac{1}{j} = \frac{1}{{j_{\mathrm{d}}}} + \frac{1}{{j_{\mathrm{k}}}} = \frac{1}{{B\omega ^{1/2}}} + \frac{1}{{j_{\mathrm{k}}}},\;\;\;B = 0.62nFC_0\left( {D_0} \right)^{2/3}\nu ^{ - 1/6}$$

Where *j* indicates current density (mA cm^−^^2^), *j*_d_ for diffusion limited current density (mA cm^−2^), *j*_k_ for kinetic current density (mA cm^−2^), *ω* for angular velocity of the disk electrode (rad s^−1^), *n* for electron transfer number (n), *F* for Faradaic constant (96485 C mol^−1^), *C*_0_ for concentration of dissolved oxygen in the 0.1 M KOH (1.2 * 10^−3^ mol L^−1^), *D*_0_ for diffusion coefficient of dissolved oxygen in the 0.1 M KOH (1.9 * 10^−5^ cm s^−1^), *ν* for kinematic viscosity of the 0.1 M KOH (0.01 cm^2^ s^−1^).

The transient open-circuit voltage decay (OCVD) measurements were taken under chopped light irradiation. The average lifetime of the photogenerated carriers (*τ*_*n*_) were obtained from the OCVD according to the equation (eq. 8):

8 $$\tau _n = - \frac{{k_{\mathrm{B}}T}}{q}\left( {\frac{{{\mathrm{d}}V_{\mathrm{oc}}}}{{{\mathrm{d}}t}}} \right)^{ - 1}$$

Where *k*_B_ is the Boltzmann constant, *T* is the Kelvin temperature (the product *k*_B_*T* is the thermal energy), *q* is the unsigned charge of an electron and d_*V*oc_/d_*t*_ is the derivative of the open-circuit voltage transient.

### Oxidation of glucose

The glucose oxidation was performed in a quartz cuvette (5 mL) containing a solution of glucose with different concentrations (0.01, 0.025. 0.05, 0.075, 0.1, 0.25, 0.5, 0.75, 1, and 2 M) and 2 mg photocatalyst in 4 mL phosphate buffer solution (0.1 M, pH 7). The photoreaction tests were done under continuous O_2_-purging with 30 min irradiation. After filteration, the colorimetric sensing of photogenerated H_2_O_2_ was analysed for the detection of glucose oxidation. For further comparison of the selectivity, the same photoreaction was carried out as glucose except replacing the glucose by 0.1 M fructose, lactose, and maltose, respectively. The effluent of CO_2_ in the closed reactor with O_2_-saturated glucose buffer solution (1 M) was tested from the calibrated gas chromatography with flame ionization detector (GC-FID, Agilent).

### Peroxidase-like activity mimic

In a typical peroxidation reaction, 200 μL of substrate (TMB, 4 mM) was added to 3.8 mL acetate buffer solution (0.1 M, pH 4) containing 3 mM H_2_O_2_ (800 μL) and 0.5 mg mL^−^^1^ photoenzymes. During the 10 min incubation at 25 °C, the kinetics of peroxidase-like activity was measured by monitoring the absorbance at 652 nm after filteration, which represents the concentration of the oxidized product of TMB. The optimized condition was modulated from the variable pH and temperatures. To measure the steady-state kinetics, the various concentrations of substrates (H_2_O_2_, 3 mM) and H_2_O_2_ (TMB, 4 mM) were used, separately. For colorimetric detection of H_2_O_2_, 200 μL of substrate of TMB (4 mM) was added to 3.8 mL acetate buffer solution (0.1 M, pH 4) containing 0.5 mg mL^−1^ photoenzyme and H_2_O_2_ with various concentrations. After incubation for 10 min in dark, the concentrations dependent blue colour at 652 nm was recorded via UV–Vis spectroscopy. The kinetic parameters were calculated using the Michaelis–Menten equation (eq. 9):

9 $$V = V_{{\mathrm{max}}}\left[ {{S}} \right]{\mathrm{/}}\left( K_{\mathrm{m}}{\mathrm{ + }}\left[ {{S}} \right] \right)$$

where [*S*] is the concentrations of substrates.

### Enzymatic cascade reaction

In a quartz cuvette, 2 mg photocatalyst was added into 2 mL phosphate buffer solution (0.1 M, pH 7) containing different concentrations of glucose. After 20 min irradiation with continuous O_2_-purging, 200 μL TMB (4 mM) and 1.8 mL acetate buffer solution (0.1 M, pH 4.0) were added into above reaction solutions, which were then incubated for another 10 min in dark. The final reaction solution was recorded by UV–Vis spectroscopy after filteration.

### Enzymatic cascade reaction in a microfluidic photoreactor

The AKCN were immobilized on the poly(dimethylsiloxane) (PDMS) wall with 3-glycidoxypropyltrimethoxysilane (GLYMO) brush, originated from the shadow-mask of lithograph defined shape and location in spatial distribution, and finally sealed with the packed slide glass. The microfluidic device was systematically set up with light source and syringe-pump connection through the silica fibers (Supplementary Figure 17a). For the redox-coupled enzymatic mimicking in separated chambers, the chip was positioned on a home-built holder to control the light irradiation on required area. The reaction volume are consist of a length of 30 mm, a depth of 0.044 mm from a cross section, and a wide of 5 mm (Supplementary Figure 17c and 17d). The enzymatic cascade reaction in microfluidic photoreactor were carried out as the same conditions as aforementioned batch process. A phosphate buffer solution (0.1 M, pH 7) containing different concentrations of glucose was pumped (12 µL min^−1^) into the microfluidic channels from the solution inlet. Meanwhile, the continuous O_2_ was pumped (12 µL min^−1^) into the flow channels from the adjacent gas inlet leading to mix with the glucose solution at cross point. As the O_2_-solution flowed into the paralleled reactive chamber, the GOx mimicking occurred on AKCN (0.25 mg chamber^−1^) under the back irradiation (seen the image of setup in Supplementary Figure 17e). For the real-time detection of glucose in the sequential processes, the TMB solution (4 mM) was pumped (12 µL min^−1^) from the downstream inlet for the continuous peroxidase-like activity in the individual chamber (0.25 mg chamber^−1^, AKCN) without irradiation. The reactive solution was spilled from the outlet and collected for the ox-TMB detection through the colorimetric method. As the same procedure without TMB injection, the concentration of H_2_O_2_ in the spilled solution was determined from DPD method. The turnover frequency (TOF) in the flow chamber and batch system was calculated based on the H_2_O_2_ generation in each duration as well as the assumption of active sites from all samples. All experiments were conducted at room temperature. The *v*_i_ values were calculated by least-squares fitting the equation (eq. 10) to the experimental data,

10 $$\left[ {{\mathrm{TMB}}} \right] = v_{\mathrm{i}}{\mathrm{ \times}} t$$

while *k*_obs_ was calculated by fitting the equation (eq. 11):

11 $$v_{\mathrm{i}} = k_{{\mathrm{obs}}}{\mathrm{ \times }}\left[ {{\mathrm{Glucose}}} \right]$$

### Computational details

Density functional theory (DFT) calculations were conducted using the Vienna Ab Initio Simulation Package (VASP)^59,60^. The Perdew-Burke-Ernzerhof (PBE) functional, which is based on the generalized gradient approximation (GGA), was used to treat the exchange-correlation energy^61^. The semi-empirical DFT-D3 method was employed to consider dispersion force^62^. The kinetic cutoff energy of 400 eV was employed for the expansion of the plane wave. A conjugate gradient algorithm was applied to relax the geometries until the forces on all the unrestricted atoms were less than 0.03 eV Å^−1^. The width of Gaussian smearing for the occupation of electronic levels is 0.2 eV. The convergence criterion for electronic structure iteration was set to be 10-4 eV. The Brillouin zone is sampled with a 5 × 5 × 3 for a (1 × 1 × 2) supercell of bulk GCN and 3 × 3 × 1 for (2 × 2) slab structure using a Monkhost–Pack scheme^63^. The relaxed structural parameters of bulk GCN are *a* = *b* = 7.124, *c* = 12.328. We used the three-layer slabs and the vacuum of 15 Å. The bottom first layer was fixed in their bulk positions, and the other top two layers were allowed to be fully relaxed. To quantitatively compare the degree of charge transfer, a Bader charge analysis has been carried out^64–66^.

### Reporting summary

Further information on experimental design is available in the Nature Research Reporting Summary linked to this article.

## Supplementary information

Supplementary Information

Reporting Summary

Source Data

## Data Availability

The data sets within the article and Supplementary Information of the current study are available from the authors upon request.
