# Supplementary material for: Modified carbon nitride nanozyme as bifunctional glucose oxidase-peroxidase for metal-free bioinspired cascade photocatalysis
Source: Nat Commun. 2019 Feb 26;10:940. doi: 10.1038/s41467-019-08731-y (PMC6391499; doi:10.1038/s41467-019-08731-y)
Supplement: Supplementary file 1 — Supplementary Information [file 41467_2019_8731_MOESM1_ESM.pdf]

Supplementary Information

Modified carbon nitride nanozyme as bifunctional glucose oxidase-peroxidase for metal-free bioinspired cascade photocatalysis

Zhang et al.

Supplementary Figures

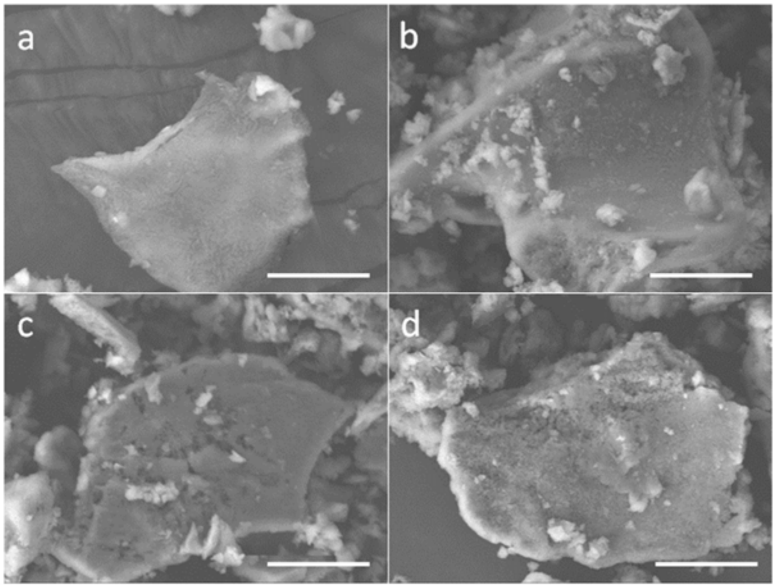

Supplementary Figure 1. Comparison of morphologiees. FESEM images of (a) GCN, (b) ACN, (c) KCN and (d) AKCN. The scale bar is 10 μm in figures.

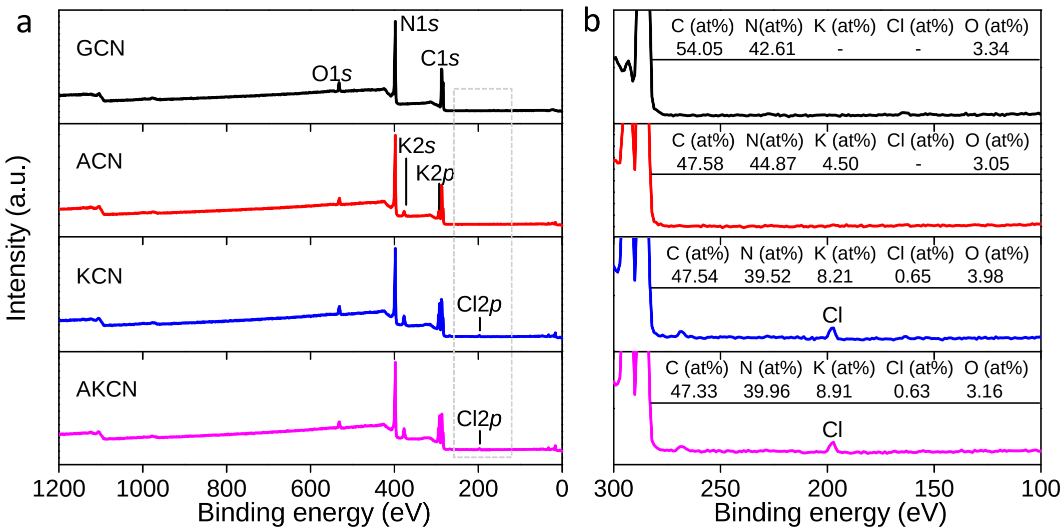

Supplementary Figure 2. The chemical states of components. (a) XPS survey spectra and (b) magnified image of panel in (a) for GCN, ACN, KCN and the AKCN. The table inset in (b) is the determined atomic concentrations of each sample based on the peak areas from the XPS survey spectra. Source data are provided as a Source Data file.

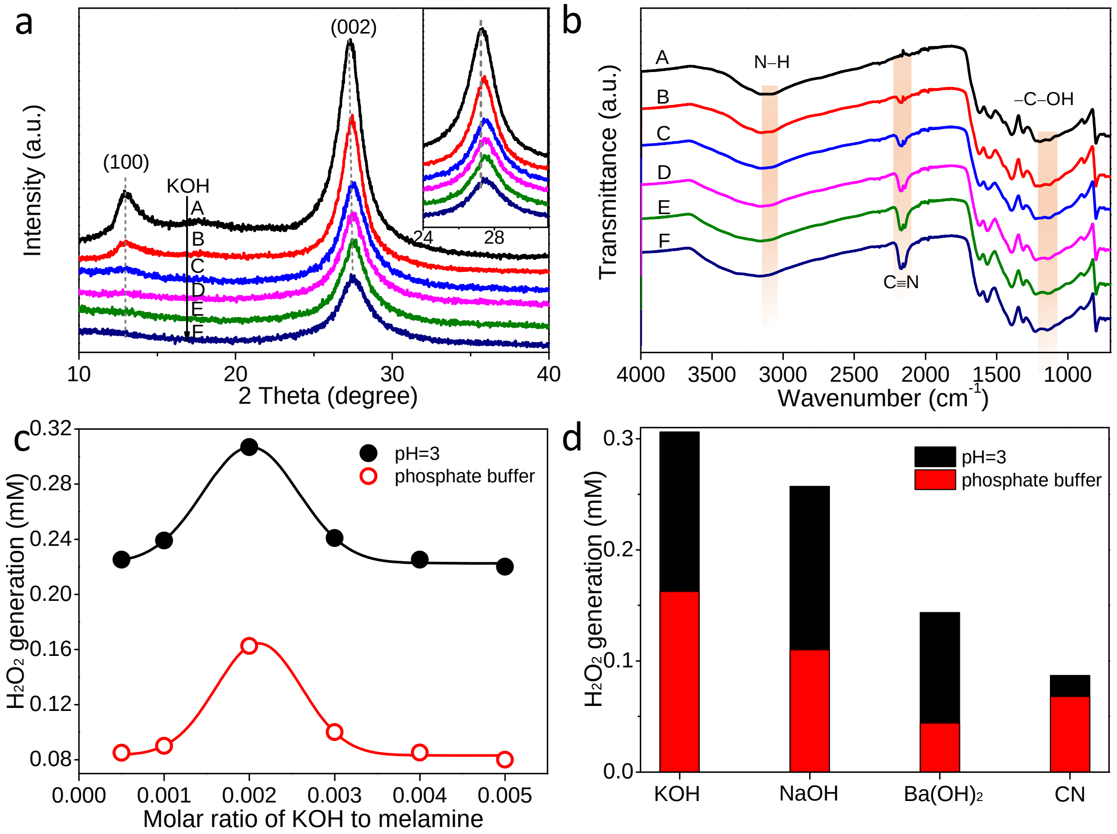

Supplementary Figure 3. Structural characterization and optimization on ACN. (a) XRD patterns and (b) FTIR spectra of ACN with different amounts of KOH. The inset in (a) is the enlarged profile of the (002) diffraction region. A–F in (a) and (b) represent GCN and the counterparts with increased molar ratio of KOH to melamine (0.0005, 0.001, 0.002, 0.003 and 0.004) in preparation. c, Photocatalytic H_2O_2 generation as a function of molar ratio of KOH to melamine in the preparation of ACN. d, Comparison of photocatalytic H_2O_2 production of GCN modified with KOH, NaOH, and $Ba(OH)_2$. Reaction conditions: the photocatalyst suspension (0.5 g L^{-1}, pH 3 or phosphate buffer) with 10 vol% EtOH in a Pyrex glass reactor under visible light illumination ($\lambda \geq 420$ nm) for 1 h, T = 25 °C. Source data are provided as a Source Data file.

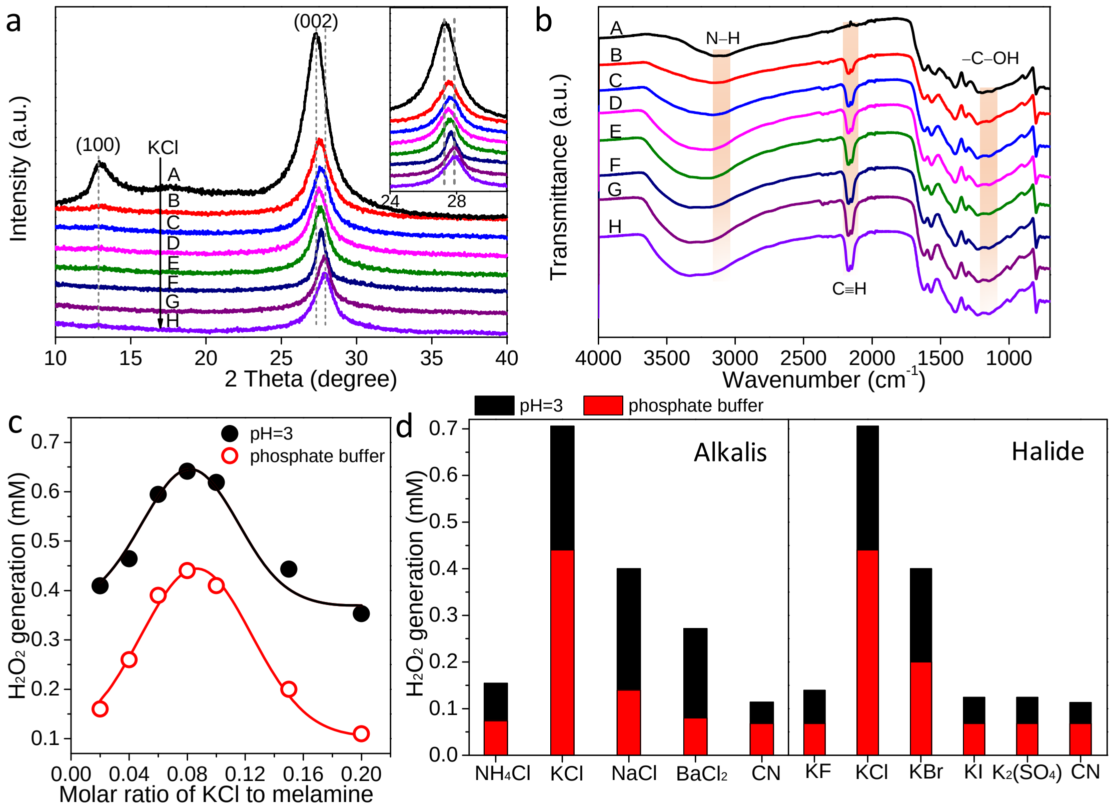

Supplementary Figure 4. Structural characterization and optimization on KCN. (a) XRD patterns and (b) FTIR spectra of KCN with different amounts of KCl. The inset in (a) is the enlarged profile of the (002) diffraction region. A–H in (a) and (b) represent GCN and the counterparts with increased molar ratio of KCl to melamine (0.02, 0.04, 0.06, 0.08, 0.1, 0.15 and 0.2) in preparation. c, Photocatalytic H_2O_2 generation as a function of molar ratio of KCl to melamine in the preparation of KCN. d, Comparison of photocatalytic H_2O_2 production of GCN modified with various alkali metal chloride, potassium halide. Reaction conditions: the photocatalyst suspension (0.5 g L^{-1}, pH 3 or phosphate buffer) with 10 vol% EtOH in a Pyrex glass reactor under visible light illumination ($\lambda \geq 420$ nm) for 1 h, T = 25 ^oC. Source data are provided as a Source Data file.

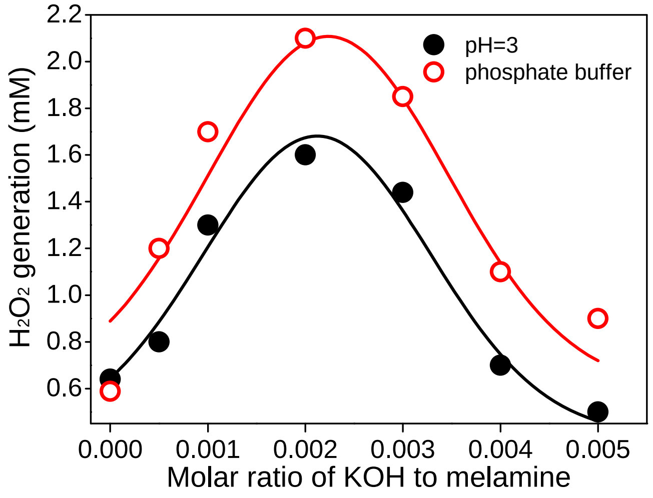

Supplementary Figure 5. Global optimization of AKCN. Photocatalytic H_2O_2 generation from optimal AKCN as a function of molar ratio of KOH in the presence of KCl (the molar ratio of KCl to melamine = 1:0.08). Reaction conditions: the photocatalyst suspension (0.5 g L^{-1}, pH 3 or phosphate buffer) with 10 vol% EtOH in a Pyrex glass reactor under visible light illumination ($\lambda \geq 420$ nm) for 1 h, T = 25 ^oC. Source data are provided as a Source Data file.

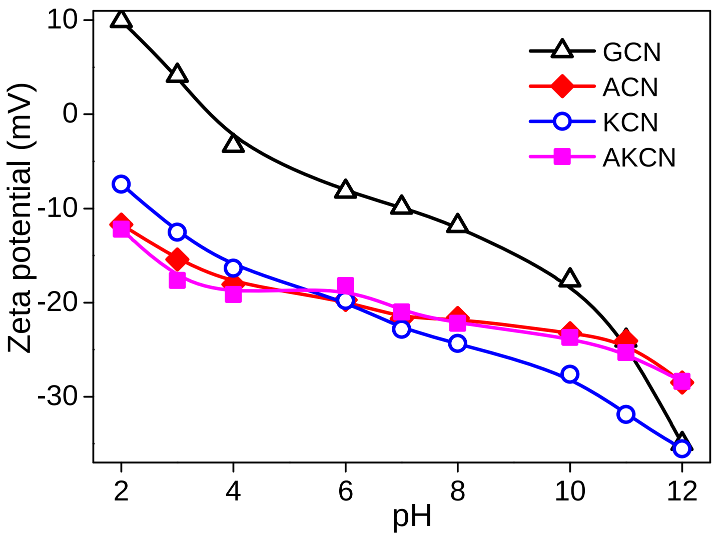

Supplementary Figure 6. Zeta potential analysis. The ξ-potentials of GCN, ACN, KCN and AKCN were obtained as a function of pH between pH 2 and pH 12. Source data are provided as a Source Data file.

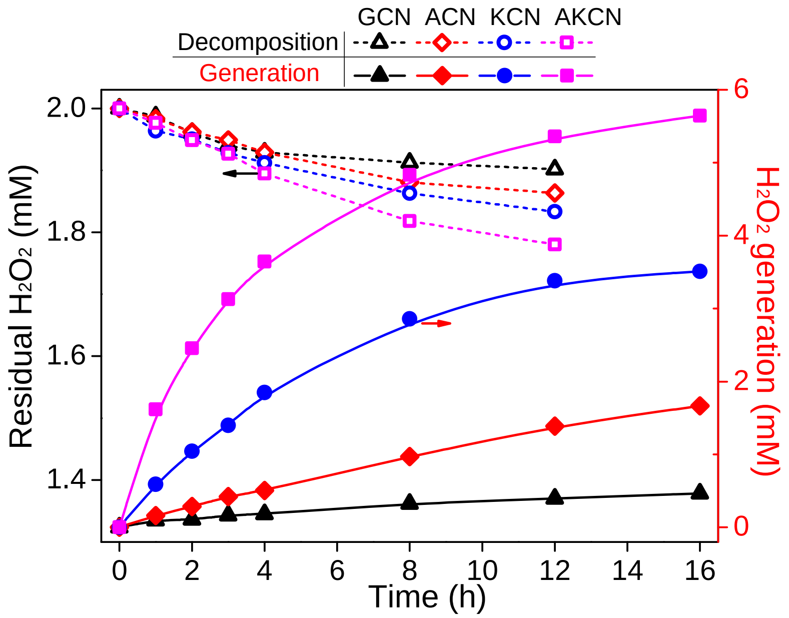

Supplementary Figure 7. The affinity of H_2O_2 on catalyst. Long-term photocatalytic evolution (right axis) and photocatalytic decomposition of H_2O_2 (left axis) among GCN, ACN, KCN, and AKCN was conducted under visible light irradiation. The photocatalytic generation of H_2O_2 was carried out under the visible-light-irradiated suspension (0.1 M phosphate buffer, pH 7) containing 10 vol% EtOH. The photocatalytic decomposition of H_2O_2 (2 mM) on GCN, ACN, KCN and AKCN was carried out in the same experimental condition except the absence of EtOH. Source data are provided as a Source Data file.

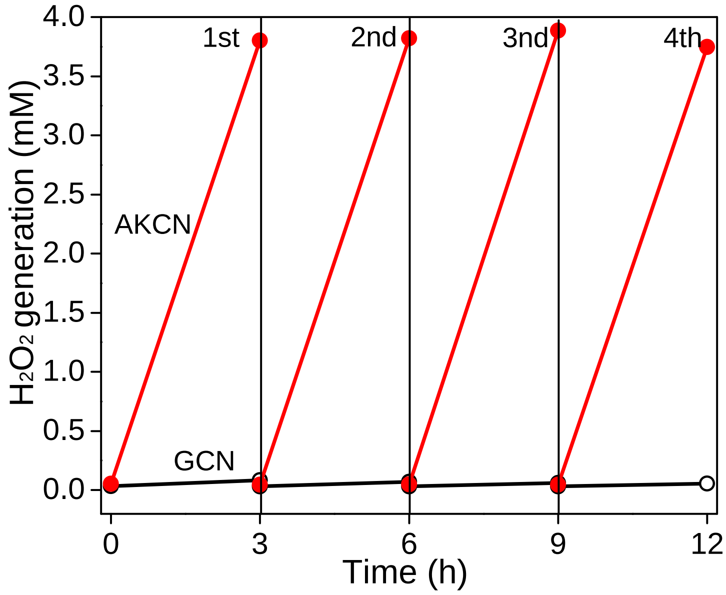

Supplementary Figure 8. Photocatalytic stability of catalysts. Repeated photocatalytic cycles of H_2O_2 generation on GCN and AKCN was conducted in the visible-light-irradiated phosphate buffer suspension (0.1 M, pH 7) containing 10 vol% EtOH. Source data are provided as a Source Data file.

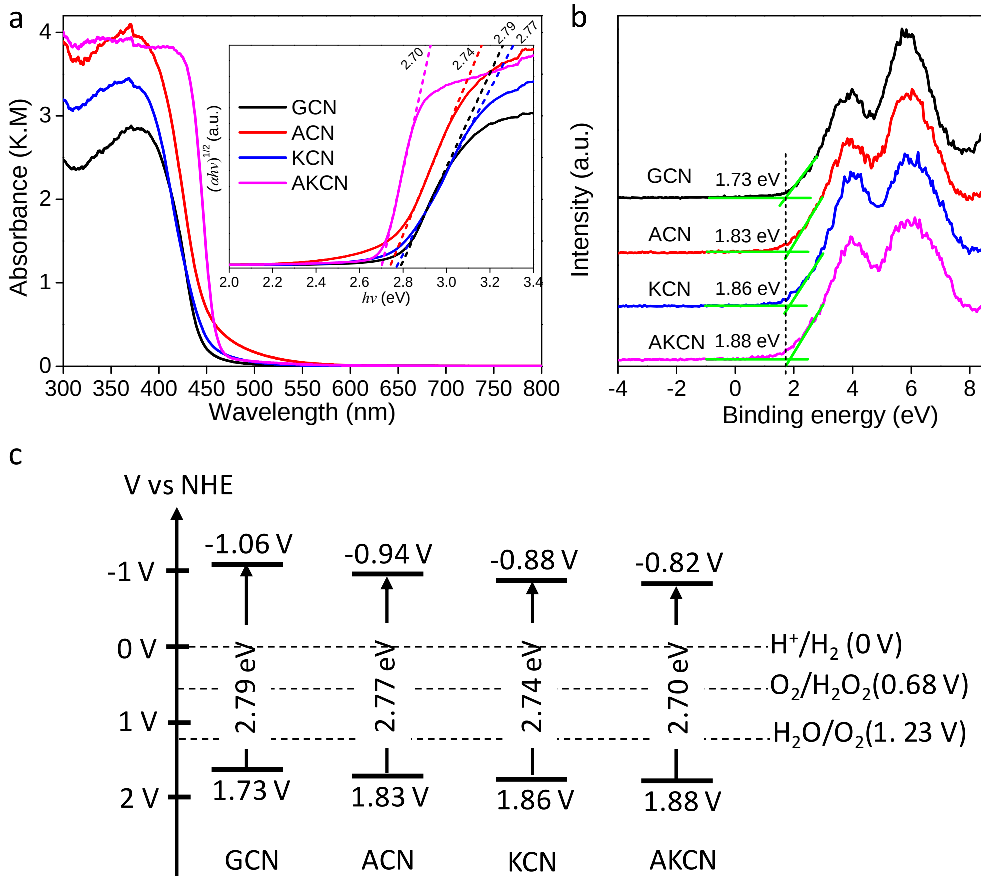

Supplementary Figure 9. Optical characterization with energy level. (a) Diffuse reflectance of UV-visible spectra (DR-UVS) with corresponding Tauc plots (inset), (b) valence band (VB) XPS, and (c) schematic illustration of the band gap structures for GCN samples. Source data are provided as a Source Data file.

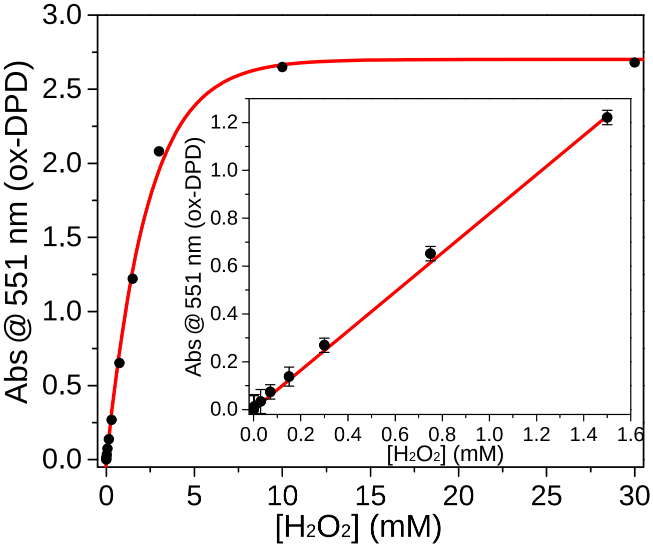

Supplementary Figure 10. Colorimetric detection of H_2O_2. The concentration calibration plot was conducted for the determination of [H_2O_2] in the colorimetric DPD method. Source data are provided as a Source Data file.

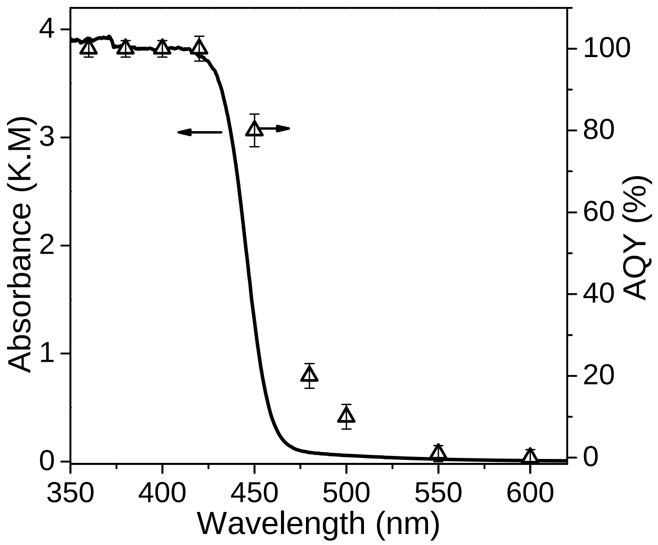

Supplementary Figure 11. Apparent quantum yield (AQY) of the glucose oxidation. The H_2O_2 production was monitored as a function of irradiation wavelength in 1 M glucose phosphate buffer suspension of AKCN (0.5 g L^{-1}), T = 25°C. The incident wavelength was adjusted with using a monochromator to select the monochromatic light from the Xe arc lamp source. Source data are provided as a Source Data file.

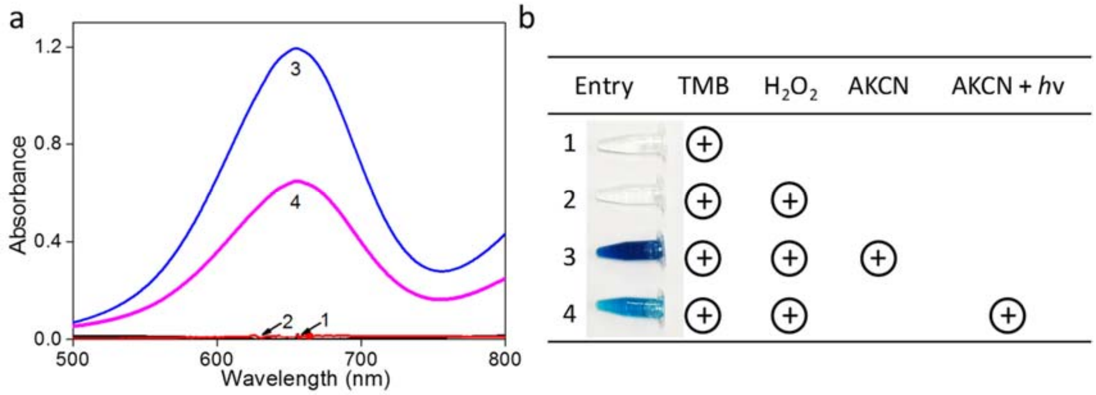

Supplementary Figure 12. The peroxidase mimicking in assayed reaction system. (a) Typical UV-visible absorption spectra along with (b) corresponding visual photograph in different condition: (1) TMB in acetate buffer solution, (2) the mixture of TMB and H_2O_2 in acetate buffer solution, (3) the mixture of TMB, H_2O_2 and AKCN in acetate buffer solution, and (4) the mixture of TMB, H_2O_2 and AKCN in acetate buffer solution after visible light irradiation ($\lambda \geq 420$ nm). [TMB] = 4 mM, [H_2O_2] = 3 mM, [AKCN] = 0.5 mg mL, acetate buffer solution (0.1 M, pH 4). It should be noted that the present experiments were performed in dark condition to prevent the over-oxidation of TMB under light irradiation (see Supplementary Figure 12b, Entry 4). Source data are provided as a Source Data file.

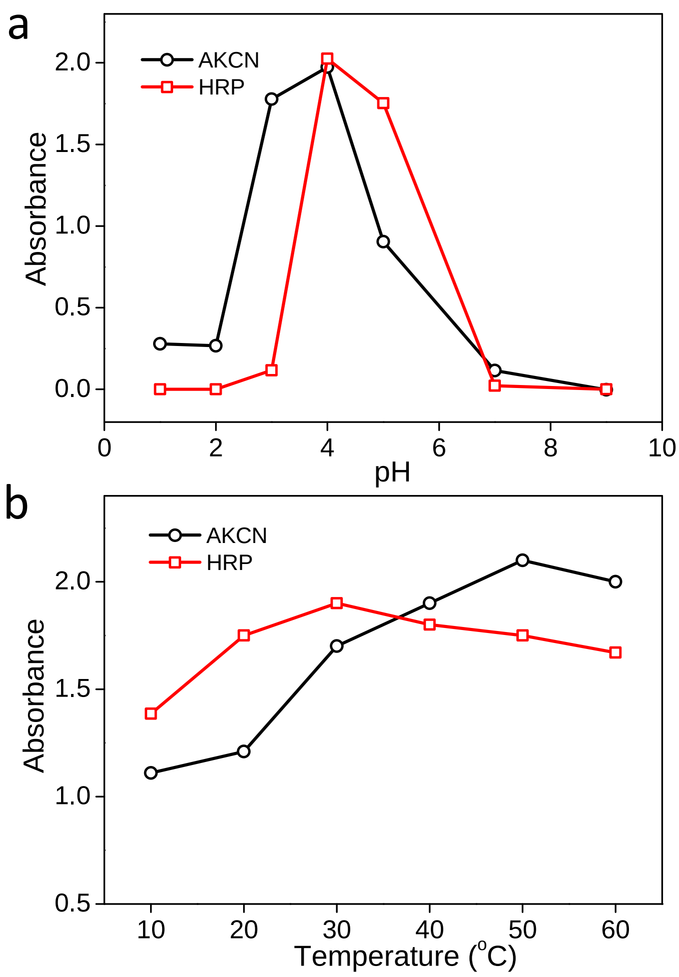

Supplementary Figure 13. Comparison of active capability between AKCN and HRP. The (a) pH and (b) temperature dependent peroxidase-like activities of AKCN (black line) and HRP (red line). Experimental condition: the buffer solution of AKCN (0.5 mg mL^{-1}) or HRP (0.05 mg mL^{-1}) with the mixture of TMB (4 mM) and H_2O_2 (3 mM) for dark incubation (10 min) in quartz cell. Source data are provided as a Source Data file.

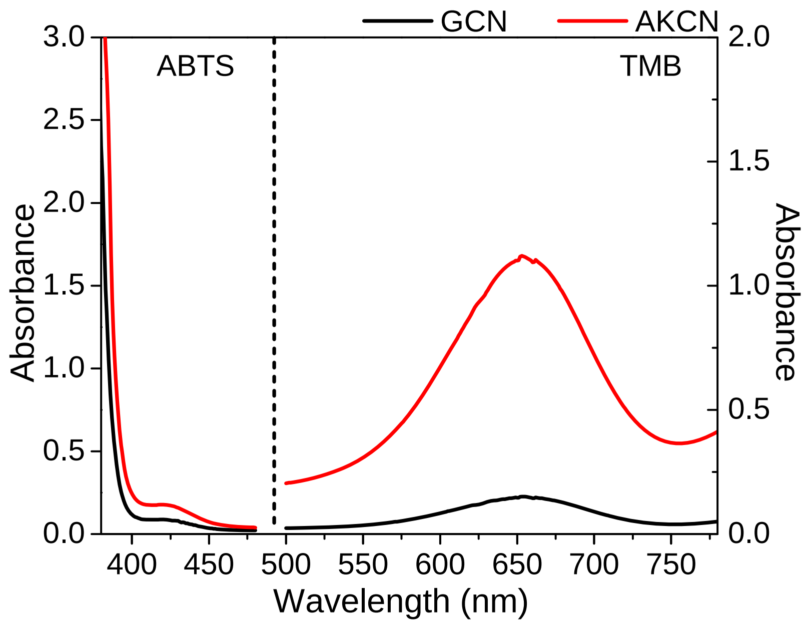

Supplementary Figure 14. Substrate specificity in peroxidase mimicking. The comparison of chromatic substrate oxidizing was performed between ABTS and TMB for GCN and AKCN. Experimental condition: the buffer solution of catalysts (0.5 mg mL^{-1}) with the mixture of chromatic substrate (4 mM) and H_2O_2 (3 mM) for dark incubation (10 min) in quartz cell. Source data are provided as a Source Data file.

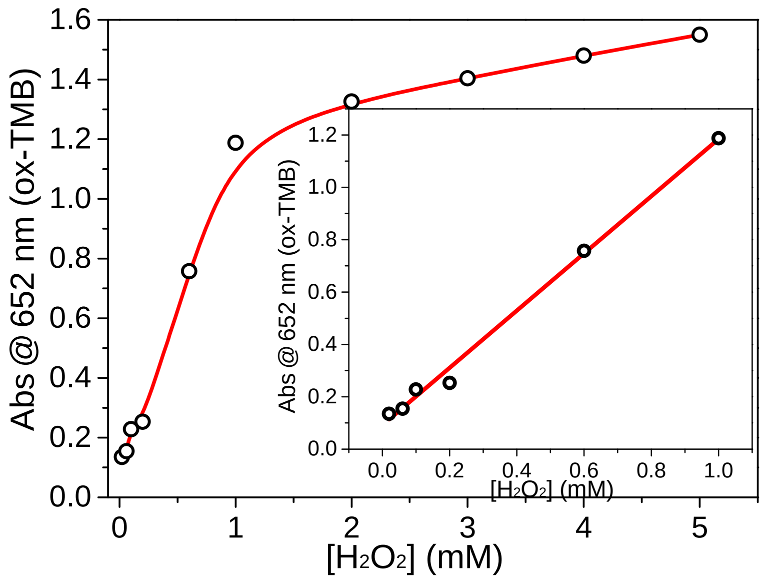

Supplementary Figure 15. TMB oxidation with respect to H_2O_2. Dependence of the absorbance at 652 nm on the concentration of H_2O_2 was conducted for colorimetric H_2O_2 detection. The inset shows the corresponding linear calibration plots. Source data are provided as a Source Data file.

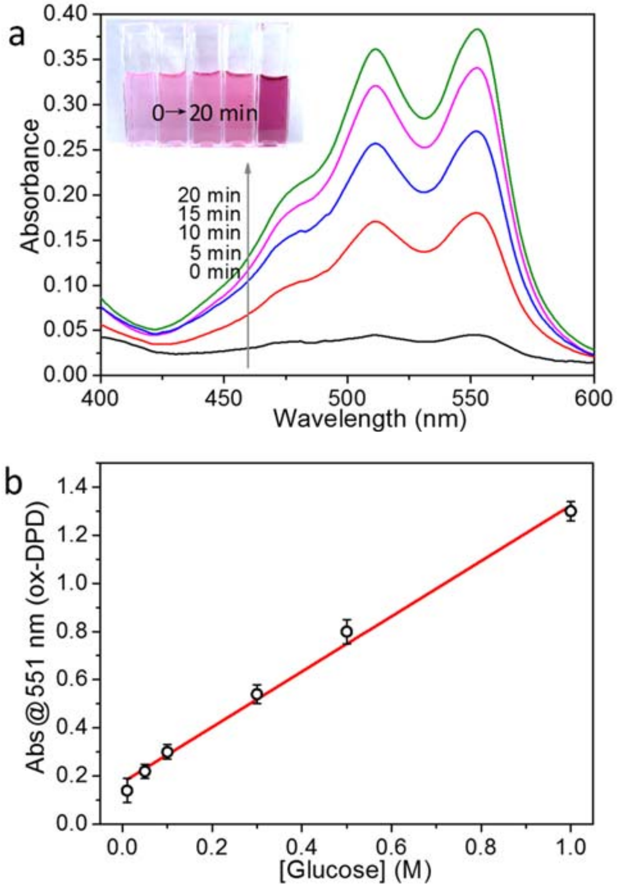

Supplementary Figure 16. Colorimetric detection of H_2O_2 against glucose in cascade reaction. a, Time-dependent absorption spectra evolution and colorimetric detection (inset) of H_2O_2 (colorimetrically monitored by DPD/POD method) in glucose solution (0.1 M) with AKCN. b, The calibration of absorbance at 551 nm for H_2O_2 detection versus the concentration of glucose in quartz cell after 20 min irradiation. Experimental condition: the phosphate buffer solution (0.1 M, pH 7) in quartz cell containing glucose solution (0.1 M) and AKCN (0.5 mg mL) under visible light irradiation ($\lambda \geq 420$ nm) with continuous O_2-purging and stirring. The error bar represents the standard deviation from the repeated experiment after three times. Source data are provided as a Source Data file.

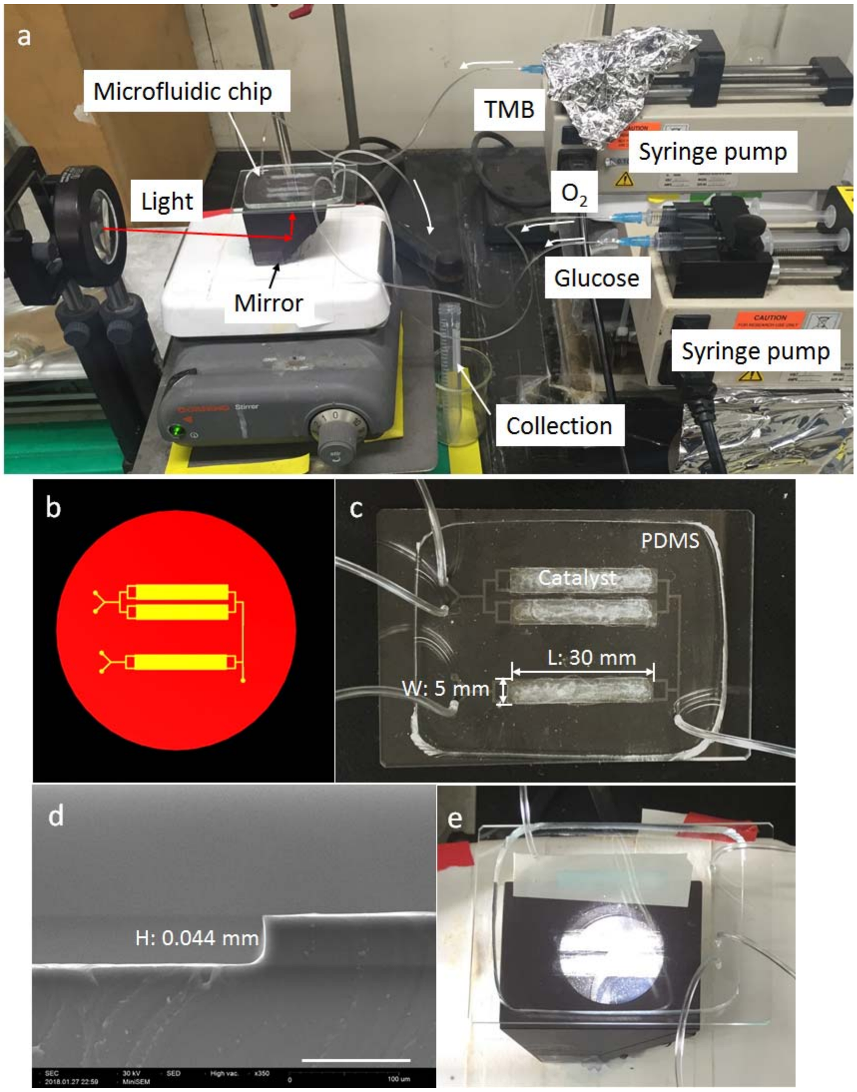

Supplementary Figure 17. Experimental set up based on microfluidics. a, Image of homemade microfluidic chip for cascade reaction. b, Photomask patterns. c, Optical image of fabricated microreactor with three inlets and one outlet. d, Cross-sectional SEM image of the reactive chamber. Scale bar was 100 μm. e, Controllable irradiation in enzymatic cascade reaction through the light shield holder.

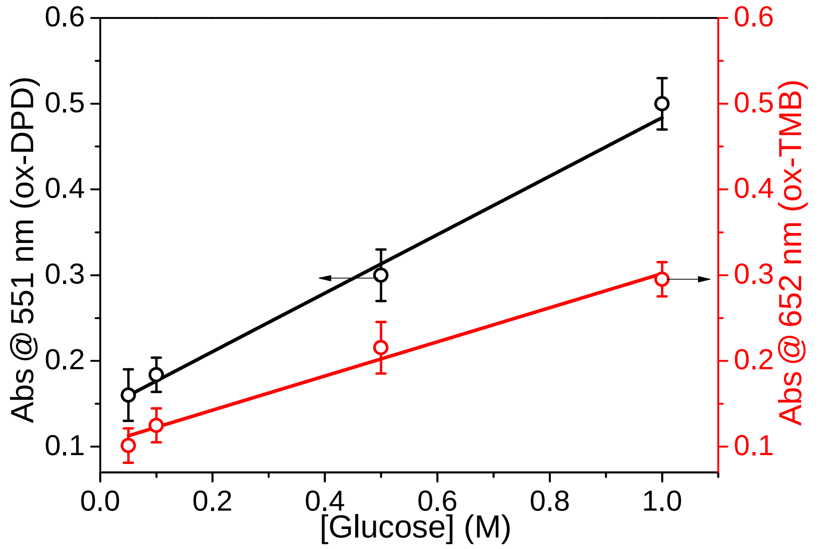

Supplementary Figure 18. Colorimetric detection of H_2O_2 against glucose in microfluidics. Dependence of the absorbance intensity of H_2O_2 (left axis) and TMB (right axis) as a function of glucose concentration was performed in microfluidic chip. The error bar represents the standard deviation from the repeated experiment after three times. Source data are provided as a Source Data file.

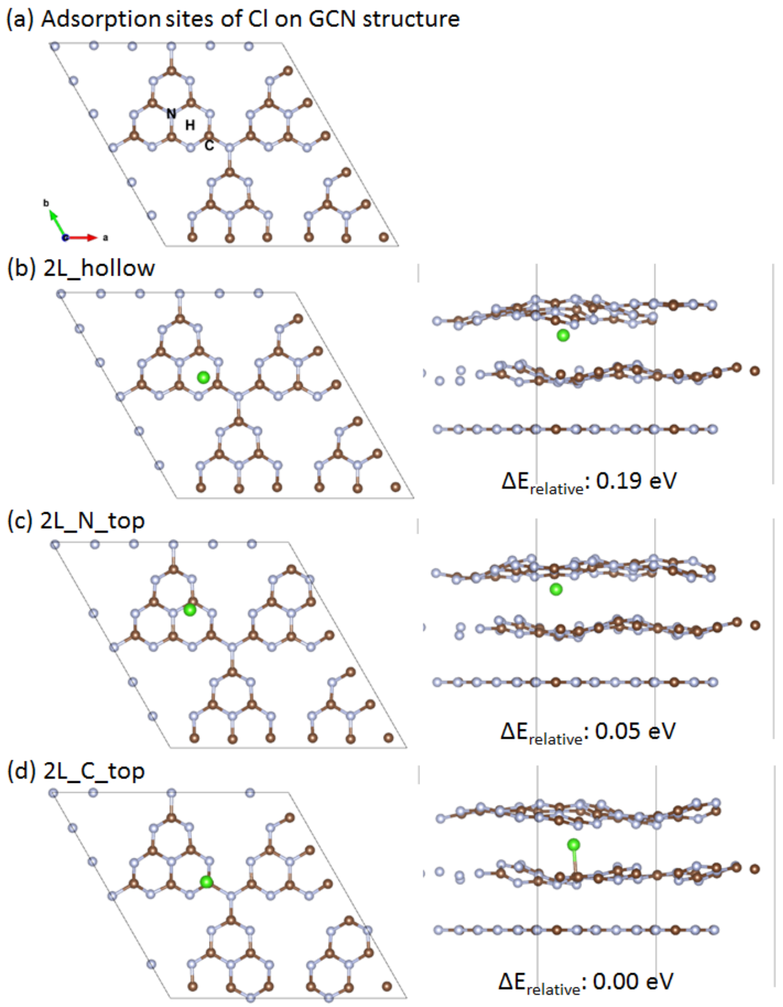

Supplementary Figure 19. Adsorption configuration of single Cl atom doped in GCN slab structure. 2L denotes adsorption site on the second layer. $\Delta E_{relative}$ represents the relative energy based on the most stable energy ($\Delta E_{relative}$ = 0.00 eV) of adsorption configuration.

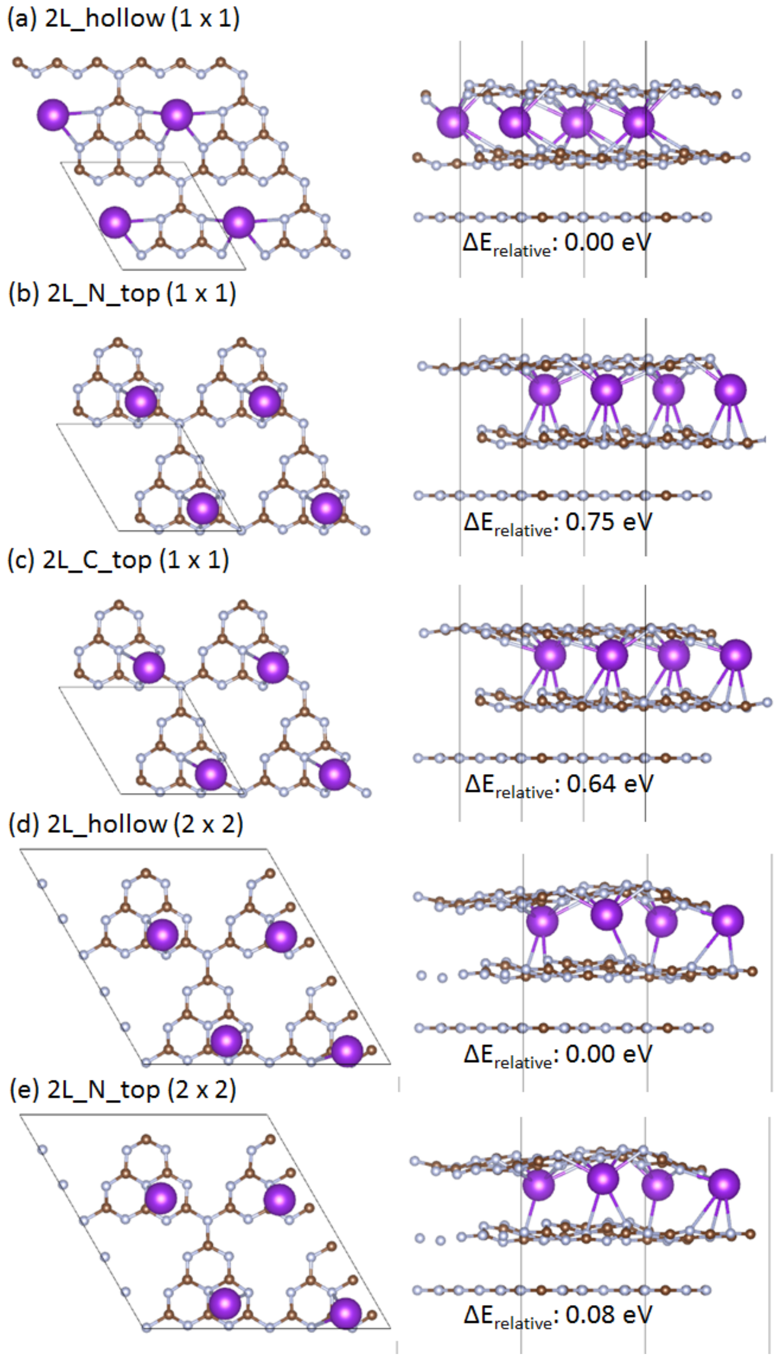

Supplementary Figure 20. Adsorption configuration of K atom doped in GCN slab structure. 2L denotes adsorption site on the second layer. $\Delta E_{relative}$ represent the relative energy based on the most stable energy ($\Delta E_{relative}$ = 0.00 eV) of adsorption configuration. For K-GCN model, we firstly calculated the doping of K atom in (1×1) slab model to expand its optimized structure to (2×2) slab model. After (1×1) calculation (a~c), the K atom migrated a lot from the initial adsorption site, especially hollow site, since the small cell size restricts the reconstruction of the first layer. Therefore, we further calculated (2×2) slab model (d~e), as a result, we found that the first layer was reconstructed flexibly and most stable K doping site is at the hollow site.

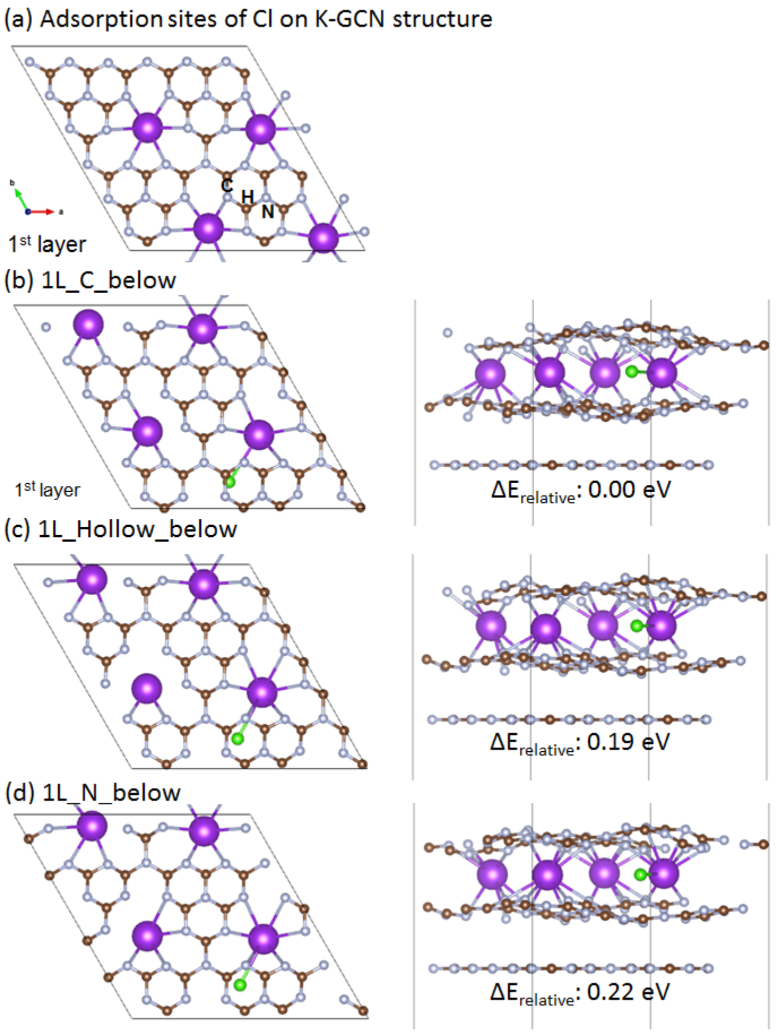

Supplementary Figure 21. Adsorption configuration of single Cl atom doped in K-GCN slab structure. 1L denotes adsorption site on the first layer. $\Delta E_{relative}$ represent the relative energy based on the most stable energy ($\Delta E_{relative}$ = 0.00 eV).

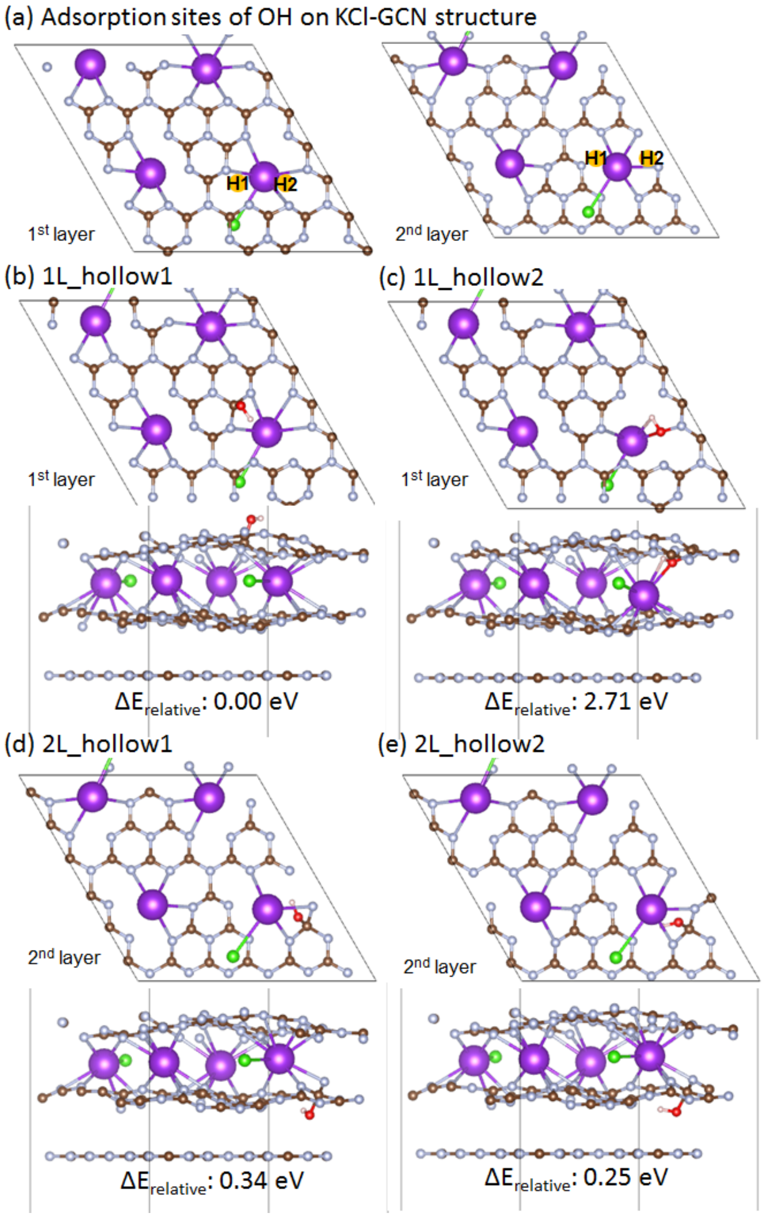

Supplementary Figure 22. Adsorption configuration of OH in KCl-GCN slab structure. 1L and 2L denotes adsorption site on the first layer and the second layer, respectively. $\Delta E_{relative}$ represent the relative energy based on the most stable energy ($\Delta E_{relative}$ = 0.00 eV).

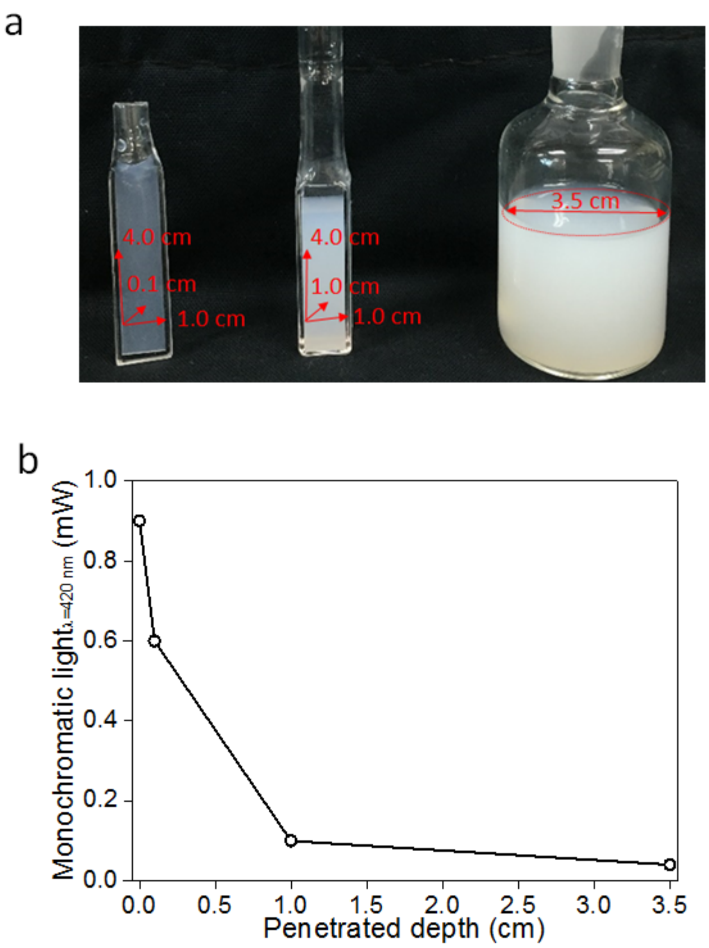

Supplementary Figure 23. Inhibition of spatial illumination in batch system. a, Image of suspension in different quartz cell ($L \times W \times H$: 4.0 cm × 1.0 cm × 0.1 cm; 4.0 cm × 1.0 cm × 1.0 cm) and batch reactor ($2R$ = 3.5 cm). b, The intensity of monochromatic light (λ = 420 nm) with respect to the penetrated depth from (a) the corresponding reactors. Source data are provided as a Source Data file.

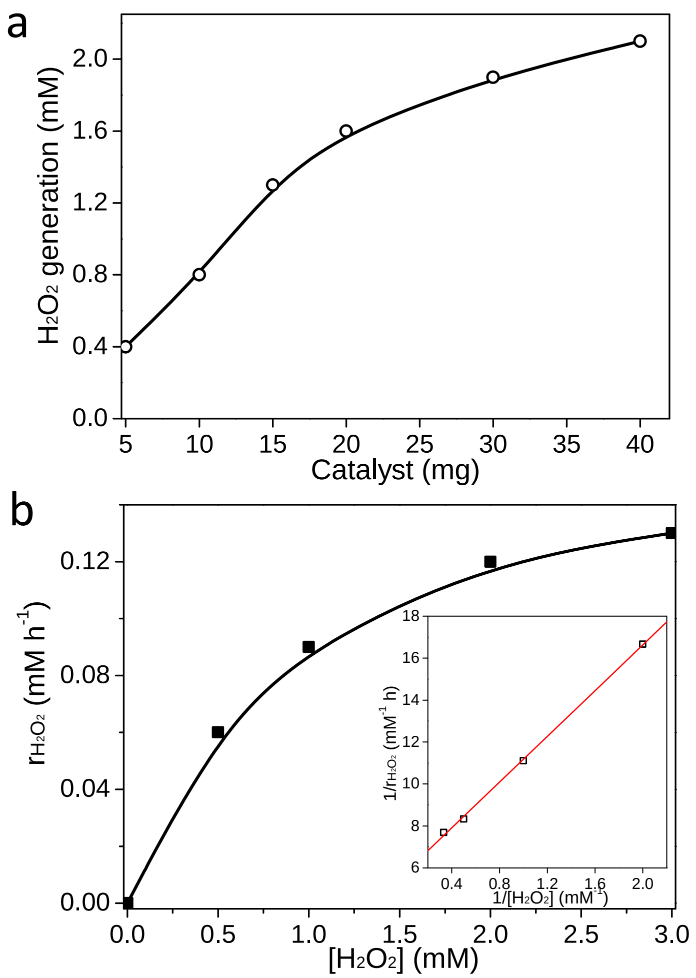

Supplementary Figure 24. The effect of diffusion on active performance in batch case. a, The active profile of H_2O_2 generation as a function of catalyst. Reaction conditions: the photocatalyst suspension (40 mL, pH 3) with 10 vol% EtOH in a Pyrex glass reactor under visible light illumination ($\lambda \geq 420$ nm) for 1 h, T = 25 ^{o}C. b, Kinetic plots of catalytic decomposition of H_2O_2. The inset diagram shows the double reciprocal plot of the data from the diagram (b). Reaction conditions: 40 mL suspension of H_2O_2 (0.5~3 mM) in a Pyrex glass reactor under visible light illumination ($\lambda \geq 420$ nm) for 1 h, T = 25 ^{o}C. It revealed that the reactive profile of H_2O_2 generation followed the zero-order kinetics as a function of the catalyst amount in batch system in (a). Before 20 mg, the reaction kinetics exhibited first-order trend from the linearly catalysis, revealing the gradually increase of active sites for sufficient reaction with saturated O_2. From the observation of moderate increase, the rate-limiting step was shifted to the process of dissolving and diffusion of H_2O_2 from catalyst surface to solution when the catalyst larger than 20 mg. Source data are provided as a Source Data file.

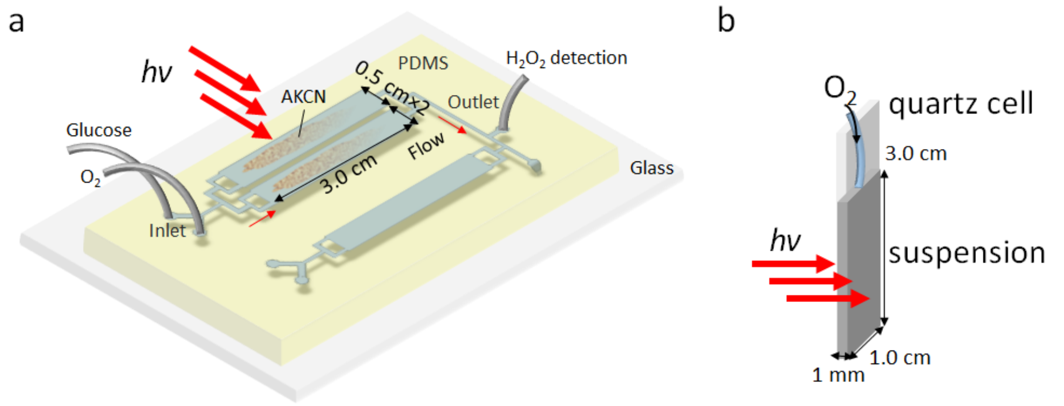

Supplementary Figure 25. The comparison of quantum efficiency between flow system and simulated batch model. Scheme of the H_2O_2 generation in a (a) microfluidic device and (b) quartz cell with O_2 purged suspension for the simulation of packed parallel chamber in microfluidic device (a).

Supplementary Tables

Supplementary Table 1 Peroxidase-mimicking nanozymes coupled with GOx for glucose detection.

Nanozyme	Minimum time required for GOx oxidation	Substrate for Peroxidase-mimic	Cascade detection (minimum time required for detection)	Detection limit	*Ref.*
$CoSe_2$/rGO	0.5 h	TMB	Glucose (0.75 h)	0.55 μM	1
Cu-Ag/rGO	0.5 h	TMB	Glucose (1 h)	3.82 μM	2
GO	1.0 h	TMB	Glucose (1 h)	1 μM	3
GO	1.5 h	TMB	Glucose (1.5 h)	16 μM	4
Fe_3O_4	0.5 h	ABTS	Glucose (0.75 h)	30 μM	5
NiO	0.5 h	TMB	Glucose (0.5 h)	20 μM	6
PVP-MoS_2	0.5 h	TMB	Glucose (1 h)	320 μM	7
Au^+	0.5 h	TMB	Glucose (0.75 h)	4 μM	8
CeO_2	0.5 h	TMB	Glucose (1 h)	3 μM	9
Co_2O_3	0.5 h	TMB	Glucose (1 h)	5 μM	10
C_3N_4	0.6 h	TMB	Glucose (1 h)	1.0 μM	11
C_3N_4 dot	0.6 h	TMB	Glucose (1 h)	0.5 μM	12
Pd-C_3N_4	0.5 h	TMB	Glucose (1 h)	1.0 μM	13
Fe-C_3N_4	1 h	TMB	Glucose (1 h)	0.5 μM	14
Carbon dot	1 h	TMB	Glucose (1 h)	0.4 μM	15
AKCN	17 s	TMB	Glucose (30 s)	0.8 μM	this work

Supplementary Table 2 Bifunctional oxidase-peroxidase mimicking nanozymes operating in cascade catalysis for colorimetric glucose detection.

Nanozyme	Substrate for Oxidase-mimic (minimum time required for H_2O_2 production)	Substrate for Peroxidase-mimic	Cascade detection (minimum time required for detection)	Detection limit	*Ref.*
Au/MOF	Glucose (4 h)	TMB	Glucose (4 h)	~8.5 µM	16
Au/V_2O_5	Glucose (0.5 h)	ABTS	Glucose (4 h)	~ 0.6 µM	17
Silica-Au	Glucose (–)	TMB	Glucose	—	18
Au@Pt	Glucose (0.5 h)	OPD	Glucose (1 h)	~0.45 µM	19
AKCN	Glucose (17 s)	TMB	Glucose (30 s)	~ 0.8 µM	this work

Supplementary Table 3 Comparison of the apparent Michaelis-Menten constant (K_m) and maximum reaction rate (V_{max}) in different system[20].

Substrate	Nanozyme	V_{max} (10^{-8} M s^{-8})	K_m (mM)
H_2O_2	AKCN	6.78	0.79
	HRP[20]	8.71	3.7
	Fe_3O_4[20]	9.78	154
TMB	AKCN	4.22	0.601
	HRP[20]	10	0.434
	Fe_3O_4[20]	3.44	0.098

Supplementary Notes

The comparison of active efficiency between batch and flow model: the whole reactive efficiency of cascade glucose detection was potentially determined from the dominated process of H_2O_2 production with respect to the limitations of photon transfer and mass transfer. The resultant microfluidics was capable of receding the unexpected above limitations to support the sustaining H_2O_2 for the subsequent TMB oxidation in cascade reaction.

Supplementary Note 1. Inhibition of photon transfer limitation (i.e., higher light utilization efficiency).

In batch system, the suspension of AKCN attenuates the intensity of incident light ($\lambda = 420$ nm) spatially from the light source to the catalyst surface through the reactor (0.9 mW to 0.04 mW) due to the summation of light scattering and absorption (Supplementary Figure 23). In contrast, the flow solution in the microfluidic chip is only hundreds of microns deep ($H = 44$ μm), allowing loss-free light to penetrate through the entire reactor for spatial illumination with higher homogeneity. The average photon intensity of optofluidics (~0.9 mW) was nine times higher than that of bulk reactor (~0.1 mW), confirming the inhibition of photon transfer limitation for superior reactive performance.

Supplementary Note 2. Inhibition of mass transfer limitation (i.e., fast reaction kinetics).

The mass transfer efficiency is mostly determined by the capability of the regents movement to the catalyst surface and the products remove through the desorption and diffusion[21]. In this case, the microfluidics with advective flow exhibits the remarkable improvement of mass transfer in catalysis because of their short molecular diffusion distances, high mass transfer rates, and large surface-to-volume ratios[22]. To help in understanding above points, we performed the series of calculation to explore the main parameters for the mass transfer predomination in chemical kinetics.

It is well known that the overall rate constant was determined by two parts, an external mass transfer from the coating catalyst to liquid phase and an intrinsic reaction rate (Supplementary Equation 1). Additionally, the diffusion also has a higher effect on fractional conversion than intrinsic rate constant in overall rate constant as batch case (Supplementary Figure 24a). Upon the generated H_2O_2 on the surface, the graphitic structure could confine the slow diffusion of H_2O_2 out of the reaction region as a result of the large diffusion length (~200 μm) of H_2O_2 for GCN[23]. However, the shorter molecular diffusion distances (44 μm in depth) in the microfluidic system can improve the mass transfer coefficient (k_m) around 5 times larger than batch case (~200 μm) from the Supplementary Equation 2. In fluidic chip, its surface to-

volume ratio (α_v) around 45454 m^{-1} was larger than that of 392 m^{-1} in batch case. Therefore, such higher surface area-to-volume ratio and mass transfer efficiency would significantly contribute for H_2O_2 generation within a few seconds than batch system, leading to the larger effect of fractional conversion on overall rate constant[24].

$$\frac{1}{k}=\frac{1}{k_i K}+\frac{1}{k_m \alpha_v} \quad (1)$$

$$k_m = D/\delta \quad (2)$$

where k_i is the intrinsic reaction rate constant, K the Langmuir adsorption coefficient, k_m the mass transfer coefficient, α_v the surface to the reactor volume, and D and δ the diffusion coefficient of H_2O_2 (1.71×10^{-9} m^2 s^{-1}) and the thickness of diffusion layer.

On the other hand, the heterogeneous Damköhler number D_{aII}, represents the ratio of the heterogeneous reaction rate at the catalyst surfaces to the diffusion from the catalyst surfaces toward bulk solution, revealing the mass transfer limitation in the microfluidics[25]. In this case, α is frequently referred to as D_{aII} and is defined for catalytic reactions (Supplementary Equation 3), which followed the Langmuir–Hinshelwood kinetics (Supplementary Equation 4) [26]. The mass-transfer coefficient can be estimated from Sherwood numbers, where Sh was considered to reach the asymptotic value of 3.66 in laminar flow (Supplementary Equation 5). According to the calculated Da_{II} number of 0.05 in our microfluidics, it proved that there is no mass transfer limitation for catalytic reaction because of the Da_{II} number is less than 0.1.[25]

$$\alpha = D_{aII} = \frac{k_i}{(k_m \times a)/K + (k_m \times a \times c_b)} \quad (3)$$

$$-r_{H_2O_2} = \frac{1}{k_i} + \frac{1}{k_i \times K \times [H_2O_2]} \quad (4)$$

$$Sh = \frac{k_m \delta}{D} \quad (5)$$

where k_i is the intrinsic rate constant (0.17 mM h^{-1}), k_m the mass-transfer coefficient, α the interfacial area per unit volume, K the Langmuir adsorption coefficient (1.08 mM^{-1}), c_b the bulk concentration of the solution (5.40 µM), [H_2O_2] the concentration of H_2O_2, D the diffusion coefficient of H_2O_2 (1.71×10^{-9} m^2 s^{-1}) and δ the thickness of diffusion layer. The k_i and K were governed from the Langmuir–Hinshelwood kinetic model (Supplementary Figure 24b) according to Supplementary Equation 4.

Supplementary Note 3. Quantitative comparison of quantum efficiency.

To identify the comparable apparent quantum efficiency (*Φ*) from the paralleled two chambers (*L* × *W* × *H*: 3.0 cm × 0.5 cm × 0.044 mm) in microfluidics, the quartz cell with similar reactive area (suspension of *L* × *W* × *H*: 3.0 cm × 1.0 cm × 1 mm) was designed to simulate the centre cross-section of reactive region on bulk reactor (Supplementary Figure 25). It is evident from the enhanced fractional conversion ($\Phi_{\text{flow}}=1.6\ \Phi_{\text{batch}}$) on flow microfluidics attributed to the advection to diffusion, the shorter mass transport length and higher total light utilization efficiency (Supplementary Equation 6).

$$
\begin{aligned}
\varphi_{flow} &= \frac{N_{mol}}{N_{photon}} = \frac{2\times n_{H2O2}\times N_A \times h\times c}{S\times P_{flow}\times t\times \lambda} \\
&= \frac{n_{H2O2}}{P_{flow}}\times k \\
&= \frac{10.1\,\mu mol}{0.9\,mW}\times k \\
&= \frac{14.4}{9}\left(\frac{n_{H2O2}}{P_{batch}}\times k\right) \\
&= 1.6\,\varphi_{batch}
\end{aligned}
\tag{6}
$$

where N_p is the total incident photons, N_e is the total reactive electrons, n_{H2O2} is the amount of H_2O_2 molecules in batch (10.1 μmol) and flow system (0.7 μmol), N_A is Avogadro constant, *h* is the Planck constant, *c* is the speed of light, *S* is the marked irradiation area (3 cm × 1 cm), *P* is the intensity of irradiation light (0.9 mW, however the average intensity for the whole activating region in bulk reactor is 0.1 mW), *t* is the photoreaction time (5 min), and *λ* is the wavelength of the monochromatic light (420 nm). *k* is the constant parameter in equation.

Supplementary References

1. Tian, X. et al. Visual and quantitative detection of glucose based on the intrinsic peroxidase-like activity of $CoSe_2$/rGO nanohybrids. *Sens. Actuators B Chem.* **245**, 221-229, (2017).
2. Darabdhara, G., Sharma, B., Das, M. R., Boukherroub, R. & Szunerits, S. Cu-Ag bimetallic nanoparticles on reduced graphene oxide nanosheets as peroxidase mimic for glucose and ascorbic acid detection. *Sens. Actuators B Chem.* **238**, 842-851, (2017).
3. Yujun, S., Konggang, Q., Chao, Z., Jinsong, R. & Xiaogang, Q. Graphene oxide: intrinsic peroxidase catalytic activity and its application to glucose detection. *Adv. Mater.* **22**, 2206-2210, (2010).
4. Lin, L. et al. Intrinsic peroxidase-like catalytic activity of nitrogen-doped graphene quantum dots and their application in the colorimetric detection of H_2O_2 and glucose. *Anal. Chim. Acta.* **869**, 89-95, (2015).
5. Wei, H. & Wang, E. Fe_3O_4 magnetic nanoparticles as peroxidase mimetics and their applications in H_2O_2 and glucose detection. *Anal. Chem.* **80**, 2250-2254, (2008).
6. Liu, Q. et al. NiO nanoparticles modified with 5,10,15,20-tetrakis(4-carboxyl pheyl)-

porphyrin: Promising peroxidase mimetics for H_2O_2 and glucose detection. *Biosens. Bioelectron.* **64**, 147-153, (2015).
7. Yu, J., Ma, X., Yin, W. & Gu, Z. Synthesis of PVP-functionalized ultra-small MoS_2 nanoparticles with intrinsic peroxidase-like activity for H_2O_2 and glucose detection. *RSC Adv.* **6**, 81174-81183, (2016).
8. Jv, Y., Li, B. & Cao, R. Positively-charged gold nanoparticles as peroxidiase mimic and their application in hydrogen peroxide and glucose detection. *Chem. Commun.* **46**, 8017-8019, (2010).
9. Jiao, X. et al. Well-redispersed ceria nanoparticles: Promising peroxidase mimetics for H_2O_2 and glucose detection. *Anal. Methods* **4**, 3261-3267, (2012).
10. Mu, J., Wang, Y., Zhao, M. & Zhang, L. Intrinsic peroxidase-like activity and catalase-like activity of Co_3O_4 nanoparticles. *Chem. Commun.* **48**, 2540-2542, (2012).
11. Lin, T. et al. Graphite-like carbon nitrides as peroxidase mimetics and their applications to glucose detection. *Biosens. Bioelectron.* **59**, 89-93, (2014).
12. Liu, S., Tian, J., Wang, L., Luo, Y. & Sun, X. A general strategy for the production of photoluminescent carbon nitride dots from organic amines and their application as novel peroxidase-like catalysts for colorimetric detection of H_2O_2 and glucose. *RSC Adv.* **2**, 411-413, (2012).
13. Jin, X. et al. A palladium-doped graphitic carbon nitride nanosheet with high peroxidase-like activity: preparation, characterization, and application in glucose detection. *Part. Part. Syst. Char.* **1700359**, (2017).
14. Tian, J. et al. Ultrathin graphitic carbon nitride nanosheets: a novel peroxidase mimetic, Fe doping-mediated catalytic performance enhancement and application to rapid, highly sensitive optical detection of glucose. *Nanoscale* **5**, 11604-11609, (2013).
15. Shi, W. et al. Carbon nanodots as peroxidase mimetics and their applications to glucose detection. *Chem. Commun.* **47**, 6695-6697, (2011).
16. Ying, H. *et al.* Growth of Au nanoparticles on 2D metalloporphyrinic metal-organic framework nanosheets used as biomimetic catalysts for cascade reactions. *Adv. Mater.* **29**, 1700102, (2017).
17. Konggang, Q., Peng, S., Jinsong, R. & Xiaogang, Q. Nanocomposite incorporating V_2O_5 nanowires and gold nanoparticles for mimicking an enzyme cascade reaction and its application in the detection of biomolecules. *Chem. Eur. J.* **20**, 7501-7506, (2014).
18. Lin, Y., Li, Z., Chen, Z., Ren, J. & Qu, X. Mesoporous silica-encapsulated gold nanoparticles as artificial enzymes for self-activated cascade catalysis. *Biomaterials* **34**, 2600-2610, (2013).
19. Liu, J. et al. Au@Pt core/shell nanorods with peroxidase- and ascorbate oxidase-like activities for improved detection of glucose. *Sens. Actuators B Chem.* **166-167**, 708-714, (2012).
20. Gao, L. *et al.* Intrinsic peroxidase-like activity of ferromagnetic nanoparticles. *Nat. Nanotechnol.* **2**, 577-583, (2007).
21. Wang N. *et al.* Microfluidic reactors for photocatalytic water purification. *Lab Chip* **14**, 1074-1082, (2014).
22. Jensen. K. *et al.* Microreaction engineering—is small better? *Chem. Eng. Sci.* **56**, 293-303(2001).

23. Liu. J. *et al.* Metal-free efficient photocatalyst for stable visible water splitting via a two-electron pathway. *Science* **347**, 970-974, (2015).
24. Lliuta I. *et al.* Two-phase flow in packed-bed microreactors: Experiments, model and simulations. *Chem. Eng. Sci.* **73**, 299-313, (2012).
25. Lei. L. *et al.* Optofluidic planar reactors for photocatalytic water treatment using solar energy. Biomicrofluidics **4**, 043004, (2010).
26. Gorges. R. *et al.* Photocatalysis in microreactors. *J. Photoch. Photobio. A* **167**, 95-99, (2004).
