# Supplementary material for: Modified carbon nitride nanozyme as bifunctional glucose oxidase-peroxidase for metal-free bioinspired cascade photocatalysis
Source: Nat Commun. 2019 Feb 26;10:940. doi: 10.1038/s41467-019-08731-y (PMC6391499; doi:10.1038/s41467-019-08731-y)
Supplement: Supplementary file 2 — Reporting Summary [file 41467_2019_8731_MOESM2_ESM.pdf]

nature research

Corresponding author(s): NCOMMS-18-18222A-Z

Last updated by author(s): 2019.01.12

Reporting Summary

Nature Research wishes to improve the reproducibility of the work that we publish. This form provides structure for consistency and transparency in reporting. For further information on Nature Research policies, see Authors & Referees and the Editorial Policy Checklist.

Statistics

For all statistical analyses, confirm that the following items are present in the figure legend, table legend, main text, or Methods section.

n/a	Confirmed	
☐	☒	The exact sample size (*n*) for each experimental group/condition, given as a discrete number and unit of measurement
☐	☒	A statement on whether measurements were taken from distinct samples or whether the same sample was measured repeatedly
☐	☒	The statistical test(s) used AND whether they are one- or two-sided<br>*Only common tests should be described solely by name; describe more complex techniques in the Methods section.*
☐	☒	A description of all covariates tested
☐	☒	A description of any assumptions or corrections, such as tests of normality and adjustment for multiple comparisons
☐	☒	A full description of the statistical parameters including central tendency (e.g. means) or other basic estimates (e.g. regression coefficient) AND variation (e.g. standard deviation) or associated estimates of uncertainty (e.g. confidence intervals)
☐	☒	For null hypothesis testing, the test statistic (e.g. *F*, *t*, *r*) with confidence intervals, effect sizes, degrees of freedom and *P* value noted<br>*Give P values as exact values whenever suitable.*
☐	☒	For Bayesian analysis, information on the choice of priors and Markov chain Monte Carlo settings
☐	☒	For hierarchical and complex designs, identification of the appropriate level for tests and full reporting of outcomes
☐	☒	Estimates of effect sizes (e.g. Cohen's *d*, Pearson's *r*), indicating how they were calculated

Our web collection on statistics for biologists contains articles on many of the points above.

Software and code

Policy information about availability of computer code

Data collection	Vienna Ab Initio Simulation Package (VASP), ChemDraw
Data analysis	Origin 9

For manuscripts utilizing custom algorithms or software that are central to the research but not yet described in published literature, software must be made available to editors/reviewers. We strongly encourage code deposition in a community repository (e.g. GitHub). See the Nature Research guidelines for submitting code & software for further information.

Data

Policy information about availability of data

All manuscripts must include a data availability statement. This statement should provide the following information, where applicable:

- Accession codes, unique identifiers, or web links for publicly available datasets
- A list of figures that have associated raw data
- A description of any restrictions on data availability

The data that support the findings of this study are available in the repository “figshare” with the identifier DOI:10.6084/m9.figshare.7593884. figshare.1499292_D8”]

Field-specific reporting

Please select the one below that is the best fit for your research. If you are not sure, read the appropriate sections before making your selection.

☐ Life sciences ☒ Behavioural & social sciences ☐ Ecological, evolutionary & environmental sciences

Behavioural & social sciences study design

All studies must disclose on these points even when the disclosure is negative.

Study description	Graphitic carbon nitride-based nanozyme that mimics both Glucose oxidase and horseradish peroxidase roles and enzymatic cascade reaction in microfluidic reactor for real-time glucose detection. The experimental study was in mixed-method of quantitative and qualitative data.
Research sample	Nanozyme was prepared from poly-condensation of melamine together with introduction of KOH and KCl in our lab. The reproducibility of preparation and active performance for samples was confirmed.
Sampling strategy	The capability of H2O2 generation and glucose oxidation was systematically conducted through the calibration equation from colorimetric detection of H2O2 and chromatic substrates.
Data collection	The data of H2O2 generation, TMB oxidation, and glucose oxidation were collected through the colorimetric method. The characterization data were obtained from the analytic instruments.
Timing	The data was collection at the interval 30 min for detect the H2O2. The incubation time was around 10 min for TMB oxidation.
Data exclusions	There was no data exclusions from the data analysis.
Non-participation	No participant dropped out.
Randomization	P.Z., S.W. and W.C. designed the materials and carried out the experiments and data analysis. D.S. and D.K. designed the microfluidic photoreactor and performed the experimental planning and analysis; A.C. and J.H. contributed to the theoretical simulation. S.L. and J.L. contributed to the rotating disk electrode measurements. All the authors participated in the discussion. P.Z. and W.C. wrote the paper.

Reporting for specific materials, systems and methods

We require information from authors about some types of materials, experimental systems and methods used in many studies. Here, indicate whether each material, system or method listed is relevant to your study. If you are not sure if a list item applies to your research, read the appropriate section before selecting a response.

Materials & experimental systems

n/a	Involved in the study	
☒	☐	Antibodies
☒	☐	Eukaryotic cell lines
☒	☐	Palaeontology
☒	☐	Animals and other organisms
☒	☐	Human research participants
☒	☐	Clinical data

Methods

n/a	Involved in the study	
☒	☐	ChIP-seq
☒	☐	Flow cytometry
☒	☐	MRI-based neuroimaging
